# WORMHOLE: Novel Least Diverged Ortholog Prediction through Machine Learning

**DOI:** 10.1371/journal.pcbi.1005182

**Published:** 2016-11-03

**Authors:** George L. Sutphin, J. Matthew Mahoney, Keith Sheppard, David O. Walton, Ron Korstanje

**Affiliations:** 1 The Jackson Laboratory, Bar Harbor, ME, United States of America; 2 Department of Neurological Sciences, University of Vermont College of Medicine, Burlington, VT, United States of America; Stockholm University, SWEDEN

## Abstract

The rapid advancement of technology in genomics and targeted genetic manipulation has made comparative biology an increasingly prominent strategy to model human disease processes. Predicting orthology relationships between species is a vital component of comparative biology. Dozens of strategies for predicting orthologs have been developed using combinations of gene and protein sequence, phylogenetic history, and functional interaction with progressively increasing accuracy. A relatively new class of orthology prediction strategies combines aspects of multiple methods into meta-tools, resulting in improved prediction performance. Here we present WORMHOLE, a novel ortholog prediction meta-tool that applies machine learning to integrate 17 distinct ortholog prediction algorithms to identify novel least diverged orthologs (LDOs) between 6 eukaryotic species—humans, mice, zebrafish, fruit flies, nematodes, and budding yeast. Machine learning allows WORMHOLE to intelligently incorporate predictions from a wide-spectrum of strategies in order to form aggregate predictions of LDOs with high confidence. In this study we demonstrate the performance of WORMHOLE across each combination of query and target species. We show that WORMHOLE is particularly adept at improving LDO prediction performance between distantly related species, expanding the pool of LDOs while maintaining low evolutionary distance and a high level of functional relatedness between genes in LDO pairs. We present extensive validation, including cross-validated prediction of PANTHER LDOs and evaluation of evolutionary divergence and functional similarity, and discuss future applications of machine learning in ortholog prediction. A WORMHOLE web tool has been developed and is available at http://wormhole.jax.org/.

## Author Summary

Identifying functionally equivalent proteins between species is a fundamental problem in comparative genetics. While orthology does not guarantee functional equivalence, the identification of orthologs—genes in different organisms that diverged by speciation—is often the first step in approaching this problem. Many methods are available for predicting orthologs. Recent approaches combine methods and filter candidate predictions by “voting”—assigning confidence to ortholog pairs based on the number of predictions by independent methods. Although voting is a heuristic, it maintains precision while increasing recall. Here we employ machine learning to optimize voting by learning which methods make better predictions and, in essence, giving those methods more votes. We present a new tool called WORMHOLE that predicts a strict subclass of orthologs called least diverged orthologs (LDOs) with a high level of functional specificity by learning features of orthology that are encoded in the patterns of predictions made by 17 constituent methods. We validate WORMHOLE using multiple measures of evolutionary divergence and functional relatedness, including community standards provided by the Quest for Orthologs consortium. WORMHOLE’s particular strength lies in predicting LDOs between distantly related species, where orthology is difficult to identify and is of critical importance for comparative biology.

## Introduction

Comparative biology has become a central strategy in the study of human biology and disease. The availability of powerful genetic tools and our ability to control experimental conditions in model organisms often allows a much more detailed examination than directly studying a process of interest in humans. In diverse areas of biology—aging, development, stem cell differentiation, behavior—highly conserved molecular features have been described in model systems, even highly evolutionarily divergent organisms, and translated into useful interventions in humans. For example, the ability to delay aging by inhibition of the Target of Rapamycin (TOR) kinase was first discovered in the single-celled budding yeast *Saccharomyces cerevisiae*, and much of the work to characterize TOR signalling has been carried out in this system (reviewed by Loewith and Hall [1]). Reduced TOR signalling has since been demonstrated to increase lifespan in a range of model systems from worms to mice (reviewed by Cornu et al. [2]). Rapamycin and other drugs targeting this system are now in clinical trials for cancer [3,4] and show promise for other age-associated diseases, including Alzheimer’s disease [5]. Aging is a particularly salient example demonstrating the power of comparative biology. Lifespan studies are much shorter, much less expensive, and therefore much more tractable in invertebrate species than in vertebrates, allowing aging studies to be carried out more rapidly, on a larger scale, and in greater molecular detail for the same resource investment. To reap the practical benefits of invertebrate models in studying the genetics of human health, it is crucial to translate molecular results from invertebrates into vertebrates.

A vital step in this translation is the identification of the gene or protein that fills the functionally equivalent role in the target vertebrate species. Since functionally equivalent proteins (FEPs) are difficult to predict directly, the most commonly used surrogate is orthology. Orthologs are genes that derive from the most recent common ancestral gene by speciation (in contrast to paralogs; genes that derive from the most recent common ancestral gene by duplication) [6]. Because orthology is defined by speciation, the evolutionary history separating orthologous genes may include other categories of evolutionary event, such as duplication, deletion, and *de novo* mutation in one or both lineages after the defining speciation event. In addition to simple one-to-one mappings, these evolutionary processes allow for one-to-many and many-to-many mappings between genes that define an orthologous group in different species. The boundaries between orthologs and non-orthologs can be difficult to discriminate based on readily measured features of genes, such as sequence composition, leading to a difficult bioinformatics problem. A subset of all orthologs are the least diverged orthologs (LDO), defined as the pair of genes within an ortholog group for two species that have accumulated the fewest mutations after speciation and duplication-post-speciation events (i.e. have ‘diverged the least’) [7]. The identification of LDOs is a sub-problem of the ortholog identification, but its solution has many desirable properties. In particular, the gene pair in an ortholog group with the least sequence divergence is the most likely to have been functionally conserved by evolution [8,9]. More divergent gene pairs are more likely to have developed novel function, particularly in gene families that have undergone numerous duplication events. In this study we focused specifically on the identification of LDOs. The idea that orthologous genes tend to be more functionally similar than non-orthologous genes is called the “ortholog conjecture”, which states specifically that orthologs are more functionally similar than paralogs. There has been recent debate surrounding this conjecture. Contrary to the ortholog conjecture, Nehrt et al. [10] found that paralogs within either humans or mice were more predictive of gene function than orthologs between humans and mice based on comparison of microarray and gene ontology (GO) data, suggesting that cellular context, rather than shared sequence, may be the primary driver of functional evolution. However, bias in GO annotations tends to favor functional similarity between paralogs [11], and subsequent studies using RNA-seq data [8] or bias-corrected GO annotations [9] support the ortholog conjecture. Specifically, Chen and Zhang [8] found that gene expression similarity between orthologs is significantly higher than between paralogs across multiple tissue types, while Altenhoff et al. [9] found that functional GO annotation similarity was higher between orthologs than paralogs, and increased weakly, but significantly, with decreased sequence divergence, even across large evolutionary distance, when the GO annotations were controlled for common biases. Thus, while orthologs and FEPs are conceptually distinct, the preponderance of evidence suggests that they are related, and in particular that identifying an ortholog as a first step toward identifying an FEP is warranted. Because protein sequence ultimately determines function, the LDO—the ortholog with the least divergence in sequence—is therefore a strong estimate of an FEP. Likewise, observing high functional similarity between genes in different species provides evidence for, but does not guarantee, shared evolutionary history.

The past decade has seen an explosion of new methodologies and tools designed to predict orthologous genes between two or more species. The majority use one of two approaches: graph-based or tree-based ortholog prediction. Graph-based algorithms begin with pairwise alignments between all protein sequences from two species to estimate evolutionary distance between each protein pair, followed by orthology prediction made using a range of clustering criteria: reciprocal best hit (e.g. OMA [12], OrthoInspector [13], and InParanoid [14]), reciprocal smallest distance (e.g. Roundup [15]), best triangular hit (e.g. COG [16] and EggNOG [17]), or Markov clustering (e.g. OrthoMCL [18]). Tree-based systems take advantage of our understanding of evolutionary relationships between species, using simultaneous alignment of sequences from many species to build phylogenetic trees and infer orthology relationships based on tree structure. Variations on this approach are employed by many popular ortholog prediction tools: Ensembl Compara [19], metaPhOrs [20], OrthoDB [21], PANTHER [22], and TreeFam [23]. Other strategies (e.g. HomoloGene [24] and Hieranoid [25]) combine aspects of both graph- and tree-based systems, progressively applying graph-based methods at the nodes of a species tree to generate more accurate ortholog predictions while maintaining the computational efficiency inherent to tree-based methods. A further alternative strategy is to directly identify genes in a target system that fills a functionally equivalent role. For example, the Isobase algorithm infers FEPs using both sequence information and functional information encoded in protein-protein interaction (PPI) networks.

Each prediction algorithm uses a different methodology, producing overlapping but distinct sets of predicted orthologs or FEPs and displaying different strengths and weaknesses in terms of performance for the particular objective of that algorithm. Several groups have combined predictions from multiple sources in “meta-tools” to improve prediction performance. Shaye and Greenwald [26] created OrthoList, a set of human-worm orthology relationships, by simply combining the predictions from four commonly used ortholog prediction tools (InParanoid, OrthoMCL, Homologene, and Ensembl Compara) to produce a system with high recall (i.e. low false negative rate) while maintaining precision (i.e. low false positive rate) when tested on a manually curated set of human-worm ortholog pairs. MetaPhOrs was constructed by collecting phylogenetic trees from seven independent sources (PhylomeDB, Ensembl, TreeFam, Fungal Orthogroups, EggNOG, OrthoMCL, and COG) and applying a common algorithm to select orthologs between species, allowing improved ortholog prediction accuracy based on cross-tree comparison [20]. The *Drosophila* RNAi Screening Center Integrative Ortholog Prediction Tool (DIOPT) reports predictions from eight ortholog databases (Ensembl Compara, Homologene, InParanoid, OMA, OrthoMCL, PhylomeDB, RoundUp, and TreeFam) and one functional database (Isobase) between six species (human, mouse, zebrafish, fruit fly, nematode, and budding yeast) and includes a confidence score based on the number of algorithms predicting each pair, and a weighted score that takes into account functional similarity based on GO term comparison [27]. The recently published Multiple Orthologous Sequence Analysis and Integration by Cluster optimization (MOSAIC) combines ortholog predictions generated by four methods (Multiparanoid, Threshold Block Aligner (TBA), six-frame untranslated BLAST-like alignment tool (BLAT), and OMA) and applies a filtration process to optimize pairwise alignment between members of each ortholog cluster [28]. Pereira et al. developed Meta-Approach Requiring Intersections for Ortholog predictions (MARIO) to aggregate four ortholog prediction methods (reciprocal best hit, InParanoid, OrthoMCL, and Phylogeny [29]) to identify high-specificity ortholog groups that were then analyzed by multiple sequence alignment and hidden Markov models to predict novel orthologs [30]. In each case, the meta-tool is shown to improve prediction performance when compared to the individual input algorithms. To date, all of these methods use the number of algorithms that predict an ortholog as a heuristic to determine the confidence of a given prediction. However, while some use sophisticated post-processing to improve performance, none take into account the individual performance of each input algorithm when assigning confidence levels to aggregate predictions.

Here we present a novel strategy in this final category of meta-tools. The WORM-Human OrthoLogy Explorer (WORMHOLE) predicts LDOs between species by employing machine learning to differentially weight the output of 17 ortholog prediction strategies. WORMHOLE falls into a subcategory of meta-tools that do not predict orthology *de novo* (others in this category include OrthoList and DIOPT), but rather integrate information from multiple sources to refine and extend predictions. Originally developed to identify orthologous genes between humans and nematodes, we have expanded the method to include six species: *Homo sapiens* (humans), *Mus musculus* (mice), *Danio rerio* (zebrafish), *Drosophila melanogaster* (fruit flies), *Caenorhabditis elegans* (nematodes), and *Saccharomyces cerevisiae* (budding yeast). WORMHOLE considers the patterns of ortholog calls of the 17 constituent algorithms and identifies signature patterns that correspond to likely LDOs. Specifically, WORMHOLE uses the genome-wide predictions of LDOs from PANTHER (PANTHER LDOs) as a set of high-confidence examples to train machine learning classifiers.

PANTHER makes *de novo* predictions of LDOs based on evolutionary relationships. We expect that rigorous statistical criteria used by any *de novo* method will necessarily miss some true LDOs, particularly in edge cases with difficult-to-parse evolutionary history or patterns of sequence divergence (e.g. duplication-post-speciation events in both lineages). Machine learning provides a principled method to extend *de novo* predictions with new data. We used the PANTHER LDOs to define positive and negative examples, but reserved judgment on genes for which PANTHER does not identify an LDO. The machine learning classifier then identified a “signature” of LDO vs. non-LDO status from the PANTHER LDO examples that can be used to infer LDO status for previously unclassified genes. WORMHOLE provides rigorous confidence scores based on how strongly the pattern corresponds to the known PANTHER LDOs. We present six findings: 1) The patterns of ortholog calls by the 17 constituent algorithms contain sufficient information to strongly predict LDOs in the reference set. This is non-trivial because, as discussed below, none of the input algorithms are designed to explicitly predict LDOs. Nevertheless they encode LDO status in the patterns of their respective ortholog predictions. 2) The use of support vector machine classifiers (SVMs) strongly improves LDO prediction over simple voting, a baseline method used in other meta-tools. 3) This enhanced prediction depends on the evolutionary distance between organisms with greater improvement for distant comparisons, e.g. between vertebrates and invertebrates. 4) The WORMHOLE SVMs expands the number of LDOs relative to the PANTHER LDO training set. The novel LDOs maintain a similar evolutionary distance distribution and Basic Local Alignment Search Tool protein (BLASTp) alignment score to the PANTHER LDO training set, indicating that the novel predictions are indeed LDOs. 5) The WORMHOLE models trained on one pair of species generalize well to other species pairs, suggesting that the WORMHOLE models are identifying information about orthology in general, and not just between particular species pairs. 6) The WORMHOLE predictions have high functional specificity by several criteria, while making significantly more LDO calls than the PANTHER LDOs used to train the models. This indicates that WORMHOLE has extracted functionally relevant information from the constituent algorithms that is complementary to the PANTHER LDOs.

## Results

### Predicting least divergent orthologs using machine learning

**Fig 1:**
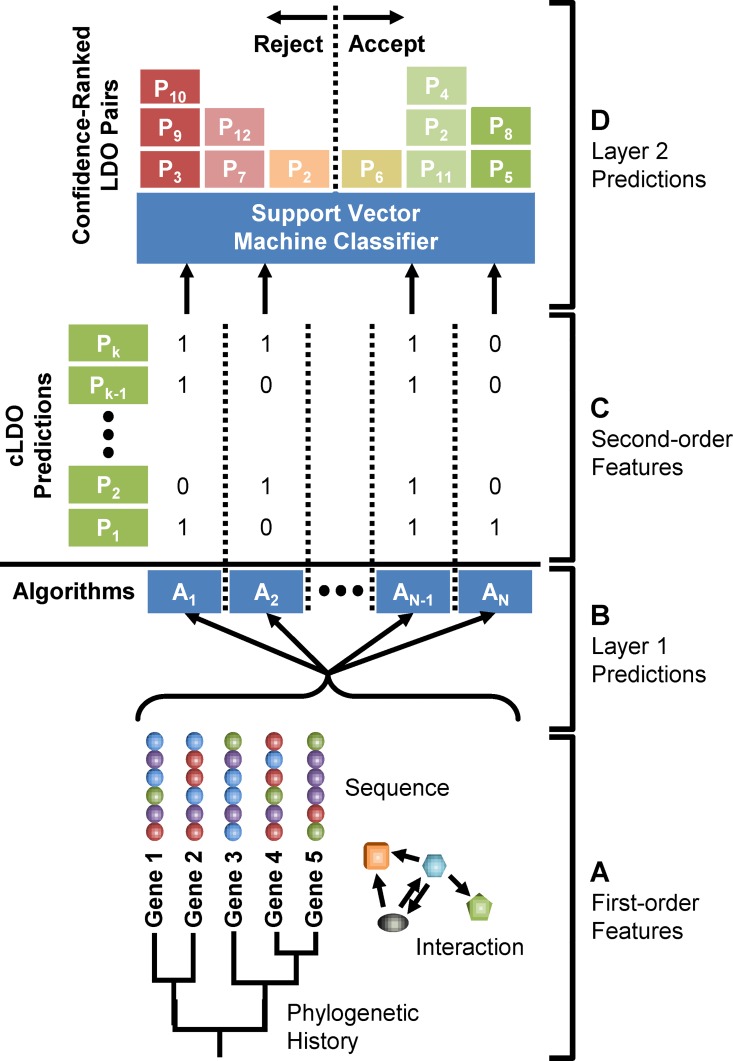
Schematic representation of the WORMHOLE LDO prediction strategy. **(A)** First-order features of gene pairs (e.g. sequence comparison, phylogenetic history, and functional interaction) are used by Layer 1 algorithms **(B)** to generate candidate LDO (cLDO) predictions, which are considered second order features **(C)**. The second-order features are used by the WORMHOLE Layer 2 methods (voting or SVMs) **(D)** to select high-confidence LDOs and filter out erroneous predictions.

Most novel ortholog prediction strategies seek to increase performance by expanding the scope or improving the quality of the underlying sequence data, or through application of a new algorithm. The wealth of ortholog prediction strategies now available opens the possibility of a *two-layer* prediction model. To conceptualize this model, consider the individual pieces of underlying biological and genetic information—gene and protein sequences, gene and protein interactions, phylogenetic relationships between species—as *first-order features* (Fig 1A). Each of the established ortholog prediction algorithms (Ensembl Compara, EggNOG, etc.) uses different combinations of these first-order features to generate predicted ortholog relationships, forming the *first layer* of prediction (Fig 1B). These algorithms generate a pool of candidate ortholog predictions, and hence candidate LDOs, that can be considered novel *second-order features* (Fig 1C). In WORMHOLE, we apply a second layer of prediction to refine these candidate ortholog predictions to directly predict LDOs (Fig 1D). This refinement is accomplished by generating a confidence score for each gene pair based on the pool of predictions and considering only those pairs that meet a minimum confidence threshold.

This multilayer approach requires three ingredients: (1) genome-wide candidate ortholog predictions (i.e. second-order features) between the species of interest generated by a selected set of first-layer algorithms, (2) a second-layer algorithm to classify each gene pair as either an LDO or not based on the second-order features, and (3) a training dataset (*reference set*) composed of well-defined examples of both LDO and non-LDO gene pairs, which is used to train and test the second layer algorithm.

To generate a genome-wide candidate pool (ingredient 1), we collected all ortholog predictions from 17 constituent algorithms between the selected species, representing a wide array of different prediction strategies. There are more than 30 databases that predict orthologous or functional relationships between species using different methodologies. In selecting algorithms to include in WORMHOLE, we sought to sample as wide a variety of prediction strategies as possible. We examined each database that we were able to locate and access online and included the 13 data sources that met the following criteria: (1) the availability for download of complete genome-wide ortholog predictions, (2) current ortholog prediction data (updated since 2010), and (3) demonstrated performance in published literature. This set includes 5 graph-based strategies, 5 phylogeny-based strategies, 2 hybrid graph- and tree-based strategies, and 1 PPI network-based strategy (Table 1). Because some projects identify multiple categories of orthologs (e.g. EggNOG-COGs and EggNOG-KOGs), these 13 sources resulted in 17 predicted ortholog datasets (*constituent algorithms*). We assembled these predictions into a common database (the *WORMHOLE database*) and call these predicted orthologous gene pairs *candidate LDOs* (cLDOs).

**Table 1:** Data sources and access dates for the 17 ortholog datasets used to build the WORMHOLE database and train the WORMHOLE SVMs.

Data Source	Ortholog Datasets	Category	Version	Access Date	Web Address
EggNOG	EggNOG-COGs,EggNOG-KOGs,EggNOG-NOGs	graph-based	4	11/28/2014	http://eggnog.embl.de/version_4.0.beta/
Ensemble	Ensembl Compara	tree-based	77	11/28/2014	http://www.ensembl.org/biomart/
Hieranoid	Hieranoid	hybrid tree- and graph-based	1	11/28/2014	http://hieranoid.sbc.su.se/
Homologene	Homologene	hybrid tree- and graph-based	68	11/28/2014	http://www.ncbi.nlm.nih.gov/homologene/
InParanoid	InParanoid	graph-based	8	11/28/2014	http://inparanoid.sbc.su.se/
Isobase	Isobase	PPI network-based	3	11/28/2014	http://groups.csail.mit.edu/cb/mna/isobase/
MetaPhOrs	MetaPhOrs	tree-based	201405	11/28/2014	http://betaorthology.phylomedb.org/
OMA	OMA	graph-based	March 2014	11/28/2014	http://omabrowser.org/oma/home/
OrthoDB	OrthoDB	tree-based	7	11/28/2014	http://cegg.unige.ch/orthodb7
OrthoMCL	OrthoMCL	graph-based	5	11/28/2014	http://www.orthomcl.org/orthomcl/
PANTHER	PANTHER	tree-based	9	11/28/2014	http://www.pantherdb.org/
RoundUp	Roundup	graph-based	2	11/28/2014	http://roundup.hms.harvard.edu/
TreeFam	TreeFam	tree-based	9	11/28/2014	http://www.treefam.org/
IsoRank	IsoRankN-PPI,IsoRankN2-GI	PPI and GI network-based	3	2/13/2014	http://groups.csail.mit.edu/cb/mna/

For a second layer algorithm (ingredient 2), we trained SVMs using the predictions of the constituent algorithms. SVMs are machine learning classifiers that take as input a set of labelled examples and a set of ‘features’ describing the examples and builds a mathematical model of each class based on the relevant information within the features. In our case, we trained SVMs on known LDO and non-LDO pairs using the orthology predictions of the 17 constituent algorithms as features. To the SVM classifier, each cLDO is represented as a signature vector of binary calls by the constituent algorithms (e.g. ‘00011011101010110’) with each digit representing the prediction made by a specific algorithm (1 = predicts orthology; 0 = does not predict orthology).

The SVMs require a reference set of known LDOs and non-LDOs to use as training data (ingredient 3). A well-defined reference set should: (1) be representative of the entire set of “true” LDOs between the species considered, (2) include only high-confidence examples, and (3) include examples of both LDO and non-LDO gene pairs. We selected the PANTHER LDO dataset as the reference set for training the SVMs. PANTHER identifies orthologous gene pairs based on species structure within algorithmically constructed phylogenetic trees. PANTHER LDOs include all one-to-one orthologs and the single least divergent gene pair in one-to-many and many-to-many ortholog groups within the broader PANTHER ortholog dataset. PANTHER LDOs consistently perform well, generating conservative predictions (i.e. fewer, more closely related ortholog pairs) when compared to other ortholog datasets using the orthology benchmarking service provided by Quest for Orthologs (QfO), a consortium that provides community standards for developing and testing orthology prediction methodology (http://questfororthologs.org/) [31]. Because the PANTHER LDO set is conservative, we anticipate that it contains strong positive examples of LDOs and that we can identify gene pairs that appear “LDO-like” with additional information not available to PANTHER. We grouped each cLDO in the WORMHOLE database into one of three classes: 1) *Known LDOs* are cLDOs that are contained in the PANTHER LDO set. 2) *Known non-LDOs* are cLDOs for which one or both genes in the pair has a predicted ortholog in the PANTHER LDO set that is *not* the other gene in the cLDO pair (i.e. is a multiple mapping for which the cLDO is not the least diverged pair). 3) *Unclassified cLDOs* are cLDOs for which neither gene in the pair has a known LDO. We trained the SVMs using only the known LDOs and known non-LDOs and reserved the unclassified cLDOs for possible novel LDO identifications. These unclassified cLDOs are exactly the edge cases where PANTHER can potentially be extended.

### Prediction performance is species-dependent

We trained an independent SVM for each pair of query and target species using the predictions made by the 17 constituent algorithms as features and the PANTHER LDOs as a reference set for classification. As a baseline aggregation strategy to benchmark the SVM performance we used simple voting—a straightforward tally of the number of constituent algorithms that predicted a cLDO—and ranked cLDOs by their vote counts. We employed nested cross-validation to ensure that the SVM models were not overfitting the training data (see Materials and Methods). A summary of the number of genes, number of ortholog pairs, and genes with multiple ortholog mappings across species is provided in Table 2, and for each species combination in S1 Table.

**Table 2:** Summary of ortholog datasets in the WORMHOLE database.

Label	# Ortholog Pairs	# Genes	# Genes with Multiple Orthologs	Mean # Orthologs per Gene	% Genes with Multiple Orthologs	% Least Evolutionarily Distant Gene Pairs
PANTHER LDOs	157,222	157,222	0	1.00	0.0%	87.2%
WORMHOLE LDOs	256,352	211,587	26,306	1.21	12.4%	78.9%
WORMHOLE RBHs	185,088	183,504	545	1.01	0.3%	88.7%
Voting	229,703	196,008	23,007	1.17	11.7%	82.0%
eggNOG-COGs	1,696,166	208,958	116,550	8.12	55.8%	12.0%
eggNOG-KOGs	9,436,066	317,809	204,709	29.69	64.4%	3.3%
eggNOG-NOGs	503,512	224,739	89,303	2.24	39.7%	41.8%
Ensembl Compara	463,438	263,162	61,521	1.76	23.4%	52.5%
Hieranoid	160,038	91,169	26,912	1.76	29.5%	60.3%
HomoloGene	191,608	157,220	11,615	1.22	7.4%	82.4%
InParanoid	489,968	229,402	44,442	2.14	19.4%	43.0%
Isobase	143,994	119,272	15,327	1.21	12.9%	63.7%
IsoRankN-PPI	372,420	316,516	38,748	1.18	12.2%	52.5%
IsoRankN2-GI	320,396	278,375	30,015	1.15	10.8%	46.9%
metaPhOrs	487,498	258,822	67,885	1.88	26.2%	44.1%
OMA	209,684	163,945	18,464	1.28	11.3%	72.4%
OrthoDB	2,095,364	230,220	118,620	9.10	51.5%	10.7%
OrthoMCL	543,490	228,942	75,884	2.37	33.1%	42.1%
PANTHER	743,310	295,836	101,785	2.51	34.4%	34.1%
Roundup	198,832	184,305	4,316	1.08	2.3%	88.0%
TreeFam	366,674	239,643	57,222	1.53	23.9%	58.2%

**Fig 2:**
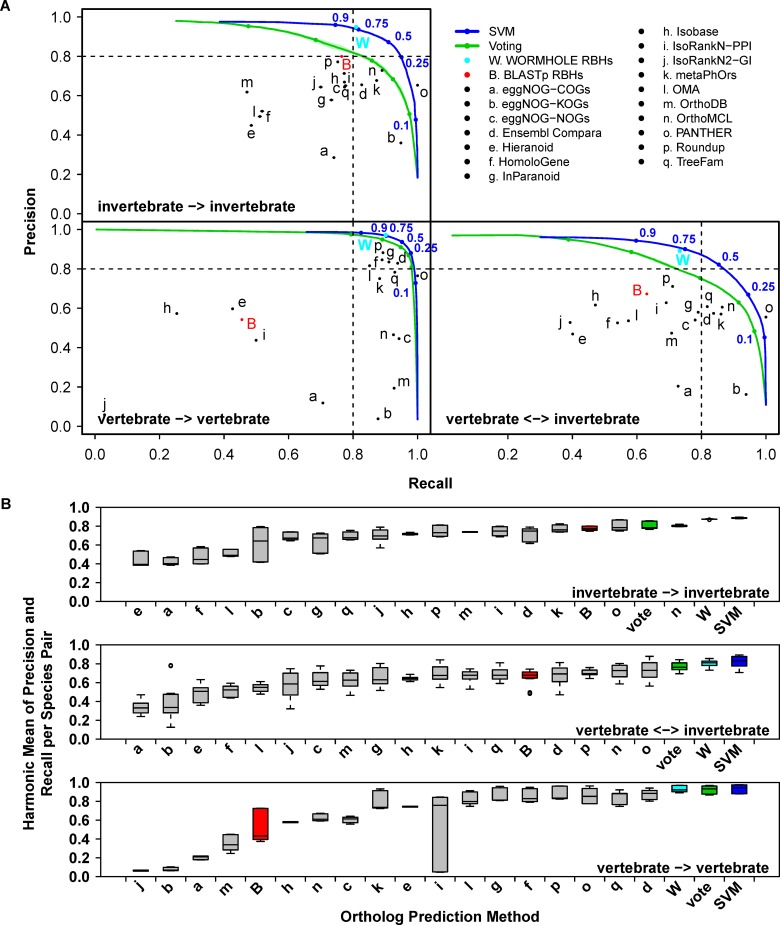
WORMHOLE SVMs improve prediction of PANTHER LDOs over constituent algorithms and voting to a degree dependent on the evolutionary separation of the compared species. **(A)** Precision-recall performance charts for PANTHER LDO predictions made between vertebrate and invertebrate species separated into categories based on evolutionary distance. Points or lines represent the mean performance of the 17 constituent algorithms (black), BLASTp reciprocal best hits (RBHs) (red), voting (green), WORMHOLE SVMs (blue), or WORMHOLE RBHs (cyan) at predicting PANTHER LDOs across the 10-folds of the outer cross-validation (see Materials and Methods). WORMHOLE RBHs are reciprocal best hits selected based on the WORMHOLE Score and are introduced later in the Results section. Error bars and colored regions represent standard error of mean for precision and recall across folds (due to the large number of gene pairs, error bars and regions are small and fall within the width of the point or line in most cases). Lines are generated by sampling the complete range of possible threshold values for each confidence score type. Color-matched points indicate the performance for specified threshold values (blue numbers) on each line. **(B)** Box and whisker plot representing the harmonic mean of precision and recall for each of the 17 constituent WORMHOLE algorithms, voting, BLASTp RBHs, WORMHOLE SVMs, and WORMHOLE RBHs when predicting PANTHER LDOs for each pair of query and target species. Ortholog prediction methods are ordered by median harmonic mean. For voting, SVMs, and WORMHOLE RBHs, values represent the maximum harmonic mean for each pair of query and target species (WORMHOLE Score ≥ 0.5).

As expected, the SVM models always outperformed the constituent algorithms and simple voting at predicting PANTHER LDOs in terms of precision (P, the fraction of predicted LDOs that are known LDOs) and recall (R, the fraction of known LDOs that are contained in the predicted LDOs) (Fig 2A and S1 Fig). This is because none of the constituent algorithms were designed to directly predict LDOs. The constituent algorithms display a wide range of performance at predicting PANTHER LDOs and none achieve as high performance as WORMHOLE at predicting PANTHER LDOs. While each algorithm performs well at the prediction task for which it was designed (e.g. prediction of orthologs from direct comparison of sequence, prediction of functional equivalence, identification of ortholog group with respect to a specific most recent common ancestor), the performance at predicting PANTHER LDOs depends on the similarity between PANTHER LDOs and the algorithm-specific design goal. PANTHER LDOs are a particularly conservative subset of ortholog predictions, and we observe that more conservative algorithms (e.g. Roundup) tend to achieve high precision and recall (Fig 2A and S1 Fig), while more permissive algorithms (e.g. eggNOG-KOGs; clusters of orthologs defined with respect to the most recent common ancestor, MRCA, for all eukaryotic species) tend to display high recall at the cost of low precision at PANTHER LDO prediction. PANTHER, by definition, has perfect recall of PANTHER LDOs (Fig 2A). The range of performance represented among algorithms is important, providing the SVM classifiers with a diverse set of features from which to discern “LDO-like” gene pairs and optimize LDO-prediction performance. The improved performance of WORMHOLE at predicting PANTHER LDOs demonstrates that WORMHOLE is able to consistently learn such structure, despite none of the constituent algorithms being designed to predict LDOs *per se*.

Identifying LDOs is of particular importance in distantly related species where evolutionary time has resulted in greater sequence divergence between orthologs, obscuring the lineal relationship between genes. In Fig 2 we examine the behavior of the SVMs as a function of the evolutionary distance between organisms. The set of species compared in WORMHOLE includes three vertebrate species (humans, mice, and zebrafish) and three invertebrate species (fruit flies, nematodes, and yeast). The three vertebrate species are substantially more closely related to each other than any vertebrate species to any invertebrate species, or any of the invertebrate species to one another. This allows LDO predictions to be grouped into those between closely related species (vertebrate-vertebrate) and more distantly related species (invertebrate-invertebrate and vertebrate-invertebrate). Fig 2A presents the performance of the SVM at predicting known LDOs as compared to each constitutive algorithm and simple voting. For each comparison the SVM has higher precision at every value of recall than simple voting or any of the constituent algorithms. Vertebrates are closely related evolutionarily; as a consequence the constituent algorithms already perform well and simple voting or the SVM yield only marginal improvement. This is ultimately due to the clarity of orthology relationships in closely related species; most orthologs are one-to-one mappings with relatively little sequence divergence. In contrast, the invertebrate species are each distantly related from each other and from the vertebrate species and the PR-curves show dramatic improvement in classification by the SVMs over voting and the constituent algorithms.

In order to normalize the outputs to make comparisons between groups, we scaled the output scores of the SVMs to the interval [0, 1] so that 0 and 1 represent the extremes of low and high prediction confidence, respectively (see Materials and Methods). We term the scaled confidence score the *WORMHOLE Score*. To allow direct comparison to our selected baseline, we similarly scaled the number of votes received by each algorithm to the *Vote Score*. A WORMHOLE or Vote Score of 0.5 is the point where the harmonic mean of precision and recall (F) is maximized. This point occurs at the “shoulder” of the PR-curve (Fig 2A) and denotes a convenient threshold of simultaneously high precision and recall. Fig 2B presents the range of F-values achieved by each constituent algorithm, simple voting, and the SVMs across species combinations. While simple voting generally outperforms the constituent algorithms, specific algorithms achieve greater performance in some cases, particularly when predicting LDOs between yeast and other species (S1 Fig). Indeed, the median F achieved by OrthoMCL between invertebrate species is 1.7% higher than simple voting (Fig 2B and S2 Table). In the vertebrate-vertebrate and vertebrate-invertebrate comparisons, simple voting achieves a median F 5.6% and 4.5% higher than the nearest constituent algorithm, respectively. In contrast to simple voting, the SVMs consistently outperform all constituent algorithms and simple voting, displaying median F 22.3%, 11.3%, and 1.4% higher than the nearest constituent algorithm at predicting LDOs between invertebrate-invertebrate, vertebrate-invertebrate, and vertebrate-vertebrate species, respectively (Fig 2B and S2 Table).

The ability of the SVM models to improve performance relative to voting appears dependent on the range of precision and recall represented in the underlying first-layer algorithms for a given species combination. Species combinations with little variation in recall in particular (e.g. human-to-zebrafish predictions, S1E Fig), result in little or no improvement in SVM performance over voting, while combinations with wide variation in both performance metrics see a much larger improvement from the SVM classifiers (e.g. human-to-worm predictions, S1E Fig).

**Fig 3:**
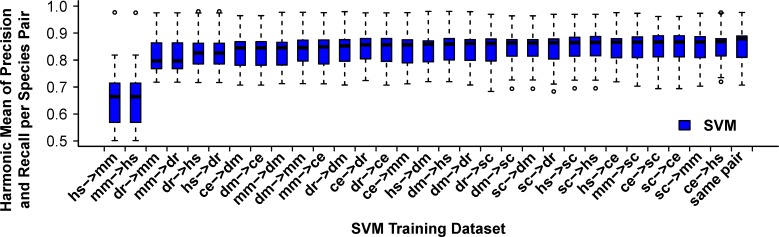
Performance of WORMHOLE SVMs generalizes across species. Box and whisker plot representing the harmonic mean of precision and recall for each WORMHOLE SVM trained on PANTHER LDOs between one pair of query and target species when applied to predict PANTHER LDOs between each other pair of query and target species. Values represent the maximum harmonic mean for each pair of query and target species (WORMHOLE Score ≥ 0.5). Training data sets are ordered by median harmonic mean. The box labelled "same pair" shows the performance of each model when applied to predict LDOs within the same species pair used to train that model (with cross-validation).

As a measure of the generalizability of the WORMHOLE SVMs, we examined the ability of a model trained on one pair of species (e.g. human-worm) to predict orthologs between each other pair of species. While optimum performance was achieved when a model was trained and tested on the same species pair, performance was surprisingly consistent across species combination (Fig 3 and S3 Table). Two species combinations were an exception to this pattern. Models trained on human-mouse and, to a lesser extent, mouse-zebrafish reference LDOs displayed reduced performance relative to the other models when applied to predict LDOs in other species combinations. Humans and mice are the most closely related species examined and have the best annotated and least divergent ortholog datasets. We speculate that the relatively poor performance of human-mouse trained SVM models at predicting LDOs in other species is a result of the limited diversity in human-mouse ortholog prediction among constituent algorithms (S1E and S1F Fig), limiting the information available to the SVM classifiers about general orthology.

**Fig 4:**
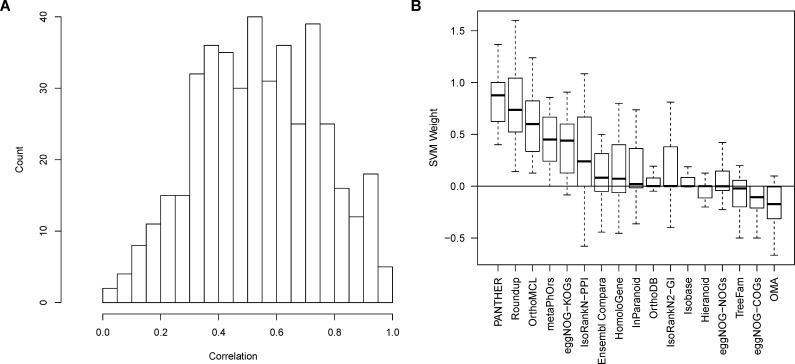
Weights given to constituent algorithm predictions by WORMHOLE SVMs are correlated across species comparisons. **(A)** Distribution of Pearson correlations between weight vectors across models trained on different pairs of query and target species show reasonable concordance across species pairs (mean = 0.54), with considerable variation (standard deviation = 0.21) indicating species-pair-specific structure in the models. **(B)** Box and whisker plot of the weights given to predictions made by each constituent algorithm by WORMHOLE SVMs trained on each pair of query and target species show that each constituent algorithm has relatively consistent weight within each species pair comparison. Note that PANTHER has the highest average weight, as expected. Ortholog prediction methods are ordered by median SVM weight.

To further examine the relationship between models trained on different pairs of species, we next examined the variation in model parameters across species combinations. Each SVM is parameterized by a set of weights assigned to predictions made by each constituent algorithm that define the classifier (see Materials and Methods). While the weights differ across species pairs, the weight vectors are correlated (mean Pearson coefficient = 0.54, standard deviation = 0.21, Fig 4A), indicating that there are global trends for particular constituent algorithms to have high or low weight across species combinations. This trend is shown in Fig 4B. As expected, PANTHER receives the highest median weight. While the constituent algorithms were developed independently, all work from similar source data and many employ related strategies to predict orthologs. As a consequence, predictions between specific tools can be highly correlated. Providing prediction data from correlated algorithms introduces redundant information that results in over-representation in the case of simple voting. The SVMs respond to correlation by proportionally reducing the weight given to the predictions from correlated algorithms. For example, predictions made by Homologene and OMA are correlated (Jaccard index = 0.46, S4 Table). We speculate that this results in OMA receiving relatively low, sometimes even negative, weight, particularly in species combinations where Homologene/OMA predictions are not well suited to predicting PANTHER LDOs. Along the same lines, WORMHOLE considers predictions from metaPhOrs, which itself is a meta-predictor incorporating sequence data from several of the other WORMHOLE constituent algorithms. As expected, metaPhOrs predictions correlate well with most of these tools, including Ensembl Compara (Jaccard index = 0.37), TreeFam (Jaccard index = 0.35), and EggNOG-NOGs (Jaccard index = 0.29), while less strongly with others (OrthoMCL; Jaccard index = 0.13) (S4 Table). Higher weight is given to metaPhOrs than any of the three highly-correlated algorithms that represent metaPhOrs source data (Fig 4B), indicating that the WORMHOLE SVMs are accounting for the correlation in assigning weights.

### WORMHOLE predictions expand LDO pool relative to the training set

**Fig 5:**
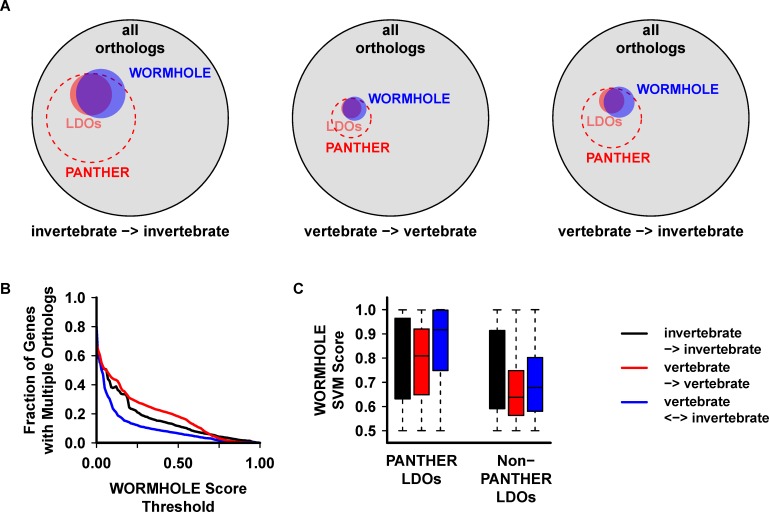
WORMHOLE SVMs reproduce the majority of PANTHER LDOs while expanding the total number of LDOs. **(A)** Venn diagrams displaying the relative number of gene pairs in PANTHER, PANTHER LDOs, WORMHOLE (WOMRHOLE score ≥ 0.5). Outer circles represent the complete set of gene pairs predicted by all of the constituent algorithms. Circle areas are proportional to the number of gene pairs in each data set. **(B)** The number of query genes with multiple LDO predictions by WORMHOLE SVMs as a function of WORMHOLE Score threshold. **(C)** Box and whisker plot representing the range of WORMHOLE Scores assigned to PANTHER LDOs or non-PANTHER LDOs (gene pairs in the WORMHOLE database but not in the PANTHER LDO reference set) within the set of genes with multiple LDO predictions by the WORMHOLE SVMs (WORMHOLE score ≥ 0.5).

WORMHOLE builds an image of what an LDO “looks like” by examining the PANTHER LDOs from the perspective of the amalgamated calls of the constituent algorithms. It then scans the collection of all cLDOs to identify novel gene pairs that fit that learned image. When applied across the genomes in question, we expect WOMRHOLE to capture an expanded set of LDOs that includes the majority of the PANTHER LDOs, as well as novel gene pairs. This is indeed what we observe (Fig 5A, Table 3). Importantly, WORMHOLE excludes a large portion of the broader PANTHER database that is not included in the PANTHER LDO set, removing the majority of the one-to-many and many-to-many gene-combinations. Importantly, the WORMHOLE classifier considers only the predictions made by the 17 constituent algorithms and is blind to the number of cLDOs corresponding to a specific query gene. As a consequence, WORMHOLE can generate multiple LDO predictions for a single query gene if there is sufficient evidence from the constituent algorithms. The number of query genes that generate multiple LDO predictions within a target species decreases as the WORMHOLE score threshold is increased (Fig 5B). Using a threshold of 0.5, WORMHOLE produces multiple LDO predictions for 12.4% of genes (Fig 5B and Table 2). Within the subset of genes with multiple LDO predictions, PANTHER LDOs receive higher WORMHOLE scores than gene pairs not in the PANTHER LDOs (Fig 5C), indicating that WORMHOLE predicts known LDOs with higher confidence than non-LDOs or novel LDOs.

**Table 3:** Comparison between PANTHER and WORMHOLD LDO sets.

Query Species	Target Species	# LDOs in PANTHER only	# LDOs in WORMHOLE RBHs only	# LDOs in PANTHER and WORMHOLE RBHs	% PANTHER LDOs Represented in WOMRHOLE RBHs	% Expansion of LDOs by WORMHOLE RBHs
all	all	28832	56698	128390	81.7%	17.7%
invertebrate	invertebrate	3260	4805	13847	80.9%	9.0%
vertebrate	vertebrate	6928	22748	63428	90.2%	22.5%
vertebrate	invertebrate	18644	29145	51115	73.3%	15.1%

To generate a high-confidence subset of the WORMHOLE LDOs that more closely matches the strict definition of an LDO, we identified WORMHOLE reciprocal best hits (RBHs). A *WORMHOLE RBH* is a predicted LDO with a WORMHOLE Score of at least 0.5 for which each gene in the pair receives the highest WORMHOLE Score when the other gene is queried (analogous to BLASTp RBHs). WORMHOLE RBHs are similar to PANTHER LDOs in that each gene in one organism will map to a single gene in the other organism. Comparing WORMHOLE RBHs to PANTHER LDOs, the WORMHOLE RBHs reproduce 81.7% of original PANTHER LDOs, but expand the total number of predicted LDOs by 17.7% (Table 3). This trend is reproduced for each comparison between vertebrates and invertebrates (Table 3). Note that in a small number of cases, multiple LDOs are predicted for a single query gene with identical WORMHOLE scores, preventing WORMHOLE from distinguishing a single RBH (Table 2). In these few cases, both predicted genes are included in the RBH category. When applied to predict PANTHER LDOs, the WORMHOLE RBHs produce similar performance to the unmodified WORMHOLE SVMs with a WORMHOLE Score of 0.75 or greater (Fig 2A).

### WORMHOLE LDOs and RBHs have low evolutionary distance

**Fig 6:**
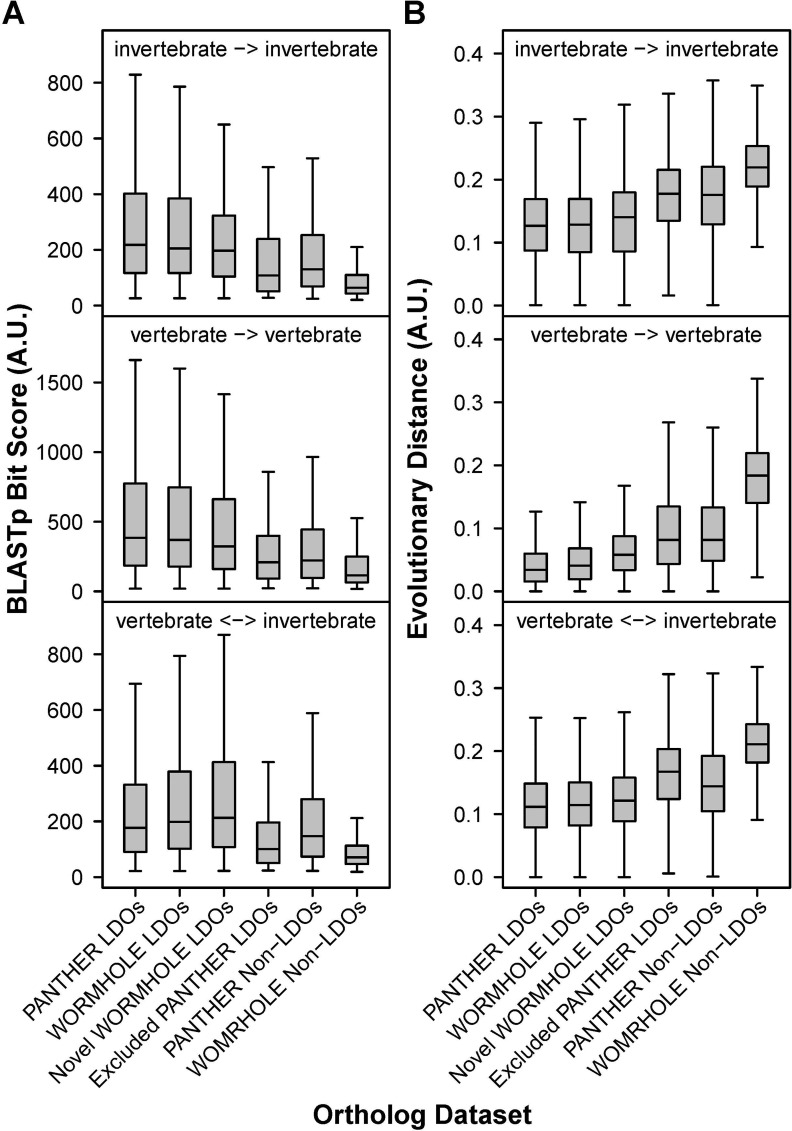
WORMHOLE identifies LDO pairs with a similar distribution of BLASTp alignment quality and evolutionary distance to the PANTHER LDOs and excludes low-scoring PANTHER LDOs. Box and whisker plots representing BLASTp Bit Score **(A)** or evolutionary distance **(B)** for alignments between longest protein isoforms for genes in each gene pair in the indicated ortholog dataset. Novel WORMHOLE LDOs are gene pairs predicted by WORMHOLE that are not present in the PANTHER LDO training set. Excluded PANTHER LDOs are gene pairs in the PANTHER LDO training set that are excluded by WORMHOLE.

By definition, the evolutionary divergence between genes in an LDO pair should be less than that between each gene in the pair and all other genes in the target genome. To evaluate the divergence of WORMHOLE LDOs and RBHs relative to PANTHER LDOs, we calculated evolutionary distance between all gene pairs for each species combination. We further examined alignment quality for each gene pair by calculating BLASTp bit scores. The set of all WORMHOLE LDOs and the set of novel LDOs predicted by WORMHOLE but not present in the PANTHER LDO training set both produce a similar distribution of evolutionary distance and bit score to the PANTHER LDOs (Fig 6). While the WORMHOLE SVMs are trained to predict LDOs based on the PANTHER LDOs, a subset of the PANTHER LDOs are excluded by the WORMHOLE SVMs. Gene pairs in this set of *excluded PANTHER LDOs* had markedly higher evolutionary distance and lower BLASTp bit scores than the WORMHOLE or PANTHER LDOs (Fig 6), indicating that the WORMHOLE SVMs specifically trimmed distantly related, low-confidence gene pairs from the PANTHER LDO dataset. A similar pattern was observed for WORMHOLE RBHs (S2 Fig). The percentage of WORMHOLE RBHs and PANTHER LDOs that identify the least evolutionarily distant gene is similar (Table 2, S1 Table). As expected, this percentage is lower for the broader category of all WORMHOLE LDOs that receive a minimum WORMHOLE Score of 0.5, which includes multiple LDO mappings for some genes (Table 2, S1 Table).

### Evaluating functional conservation

**Fig 7:**
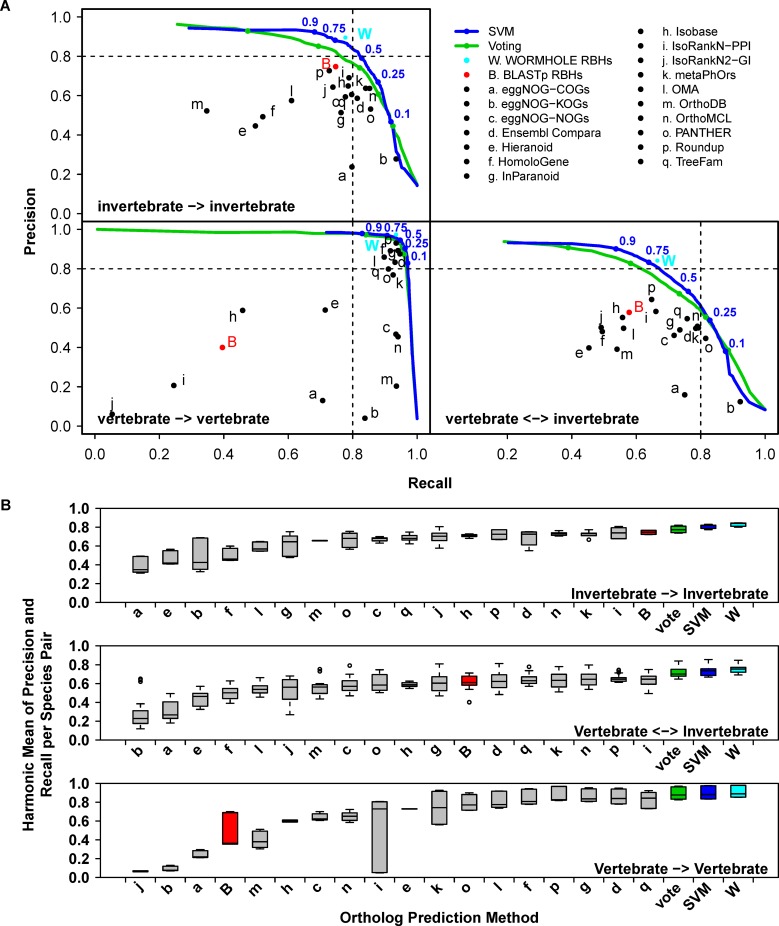
WORMHOLE SVMs improve prediction of FOSTA FEPs over constituent algorithms and voting to a degree dependent on the evolutionary separation of the compared species. **(A)** Precision-recall performance charts for FOSTA FEP predictions made between vertebrate and invertebrate species separated into categories based on evolutionary distance. Points or lines represent the mean performance of the 17 constituent algorithms (black), BLASTp reciprocal best hits (RBHs) (red), voting (green), WORMHOLE SVMs (blue), or WORMHOLE RBHs (cyan) at predicting FOSTA FEPs. Lines are generated by sampling the complete range of possible threshold values for each confidence score type. Colored points indicate the performance for specified threshold values (blue numbers) on each line. **(B)** Box and whisker plot representing the harmonic mean of precision and recall for each of the 17 constituent WORMHOLE algorithms, voting, BLASTp RBHs, WORMHOLE SVMs, and WORMHOLE RBHs when predicting FOSTA FEPs each pair of query and target species. Ortholog prediction methods are ordered by median harmonic mean. For voting and SVMs, values represent the maximum harmonic mean for each pair of query and target species (WORMHOLE Score ≥ 0.5).

Orthology is an evolutionary concept and does not necessarily imply that a pair of genes will be functionally related. However, orthologous genes, and in particular LDOs, are often functionally similar or equivalent, and ortholog prediction is commonly used as a starting point for identifying the gene or genes in a new species that fill an equivalent functional role as a gene in another species where the role is known. To assess the ability of WORMHOLE to identify functionally-related ortholog pairs, we measured the performance of the WORMHOLE SVMs at predicting Functional Orthologs from Swissprot Text Analysis (FOSTA) FEP pairs. The FOSTA database contains high confidence FEPs based on text analysis of Swiss-Prot annotations and thus represents an assessment of functional equivalence at a high level of manual curation by experts [32]. Voting improves prediction of FOSTA FEPs relative to the constituent algorithms, with SVMs giving an additional improvement in precision, recall, and harmonic mean of precision and recall (Fig 7). As observed in the ortholog reference dataset, WORMHOLE adds almost no benefit to FEP predictions between closely related species, while performance is greatly improved in more distantly related species (Fig 7B and S3 Fig). In FEP prediction between vertebrate species (Fig 7A), and predictions between humans and mice in particular (S5E and S5F Fig), many of the first-layer algorithms produce nearly perfect performance, leaving no room for improvement. In contrast, prediction of FEPs between invertebrate species, or between vertebrates and invertebrates, receives a substantial benefit from the SVM models relative to simple voting, improving both precision and recall by more than 5% in most cases and more than 10% for certain species combinations (S3 Fig). Performance statistics for WORMHOLE, voting, and each constituent algorithm at predicting FOSTA FEPs is provided in S5 Table.

**Fig 8:**
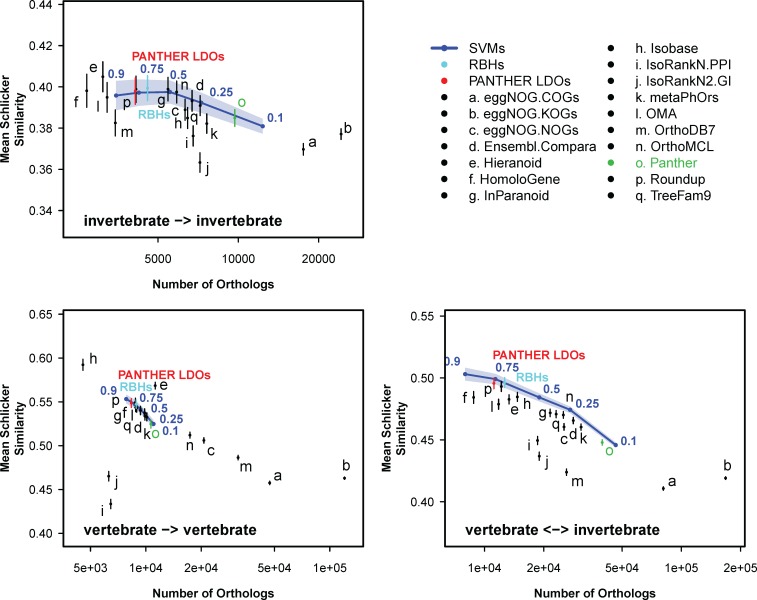
WORMHOLE SVMs produce an expanded set of LDOs while maintaining functional similarity relative to PANTHER LDOs. Conservation of GO term annotation between genes in each gene pair is plotted against the number of gene pairs contained with each dataset for PANTHER (green point), all other constituent algorithms (black points), PANTHER LDOs (red points), WORMHOLE SVMs (blue lines), and WORMHOLE RBHs (cyan points). Points or lines indicate mean, and error b ars or colored regions represent 95% confidence intervals, for Schlicker similarity in GO terms between genes (see Materials and Methods).

The QfO consortium provides a set of tools for benchmarking ortholog prediction datasets. One of these tools calculates gene ontology (GO) term conservation between gene pairs, an established metric of functional relatedness [33]. We used this service to assess the average functional relatedness between WORMHOLE-predicted LDOs as compared to predictions made by each of the constitutive algorithms and to PANTHER LDOs across the six examined genomes. WORMHOLE consistently maintained a similar level of functional relatedness between predicted gene pairs, but identified more gene pairs, as compared with the PANTHER LDOs (Table 4 and Fig 8). In invertebrate-invertebrate comparisons, WORMHOLE achieves nearly identical GO term conservation scores to PANTHER LDOs. In the vertebrate-vertebrate and vertebrate-invertebrate comparisons, WORMHOLE functional conservation is slightly decreased relative to PANTHER LDOs, but is higher than all methods that call a similar number of pairs. A similar result holds when comparing enzyme classification numbers (EC), which depend strictly on the catalyzed chemical reaction, between enzyme LDO pairs (Table 4, S4 Fig). The WORMHOLE RBHs receive similar functional relatedness and enzyme conservation scores to the PANTHER LDOs–and higher mean scores in invertebrate comparisons–while generating substantially more LDO predictions (Table 4, Fig 8, S4 Fig). A third measure evaluates the discordance between species and gene phylogenetic trees based on uploaded ortholog pairs [33]. Similar to GO term conservation, WORMHOLE expands the number of represented gene trees while maintaining low species-gene tree discordance and limiting the number of gene trees that do not match the phylogenetic structure of the species tree (S5 Fig).

**Table 4:** Gene ontology (GO) term and enzyme classification (EC) similarity for predictions by PANTHER LDOs, WORMHOLE SVMs, and WORMHOLE RBHs. SVMs and RBHs datasets contain gene pairs with WORMHOLE Score ≥ 0.5.

Dataset	Query Species	Target Species	# LDOs	GO Term Conservation(Schlicker Similarity)		# Enzyme LDOs	EC Conservation(Schlicker Similarity)	
				Mean	95% Conf.		Mean	95% Conf.
PANTHER LDOs	all	all	23600	0.497	0.0030	5934	0.988	0.0019
WORMHOLE SVMs	all	all	33865	0.486	0.0024	8132	0.977	0.0023
WORMHOLE RBHs	all	all	26082	0.497	0.0028	6521	0.984	0.0021
PANTHER LDOs	invertebrate	invertebrate	4111	0.398	0.0064	607	0.985	0.0069
WORMHOLE SVMs	invertebrate	invertebrate	5545	0.398	0.0056	836	0.977	0.0070
WORMHOLE RBHs	invertebrate	invertebrate	4569	0.399	0.0061	684	0.981	0.0069
PANTHER LDOs	vertebrate	vertebrate	8335	0.549	0.0050	2654	0.998	0.0013
WORMHOLE SVMs	vertebrate	vertebrate	9354	0.541	0.0047	2820	0.997	0.0018
WORMHOLE RBHs	vertebrate	vertebrate	8865	0.547	0.0048	2748	0.997	0.0017
PANTHER LDOs	vertebrate	invertebrate	11154	0.496	0.0043	2673	0.979	0.0037
WORMHOLE SVMs	vertebrate	invertebrate	18966	0.484	0.0032	4476	0.965	0.0037
WORMHOLE RBHs	vertebrate	invertebrate	12648	0.496	0.0040	3089	0.973	0.0039

The combined ability of WORMHOLE to improve FEP prediction and expand the pool of LDOs while maintaining functional relatedness shows that, despite non-one-to-one mapping of genes, WORMHOLE predictions are well tuned to gene function. This is demonstrated by the more restricted WORMHOLE RBHs, which maintain identical, or slightly better, functional relatedness to PANTHER LDOs while generating a larger pool of predicted LDOs. This implies that the WORMHOLE SVMs are sensitive to gene function.

### Novel predicted LDOs are high quality candidates

To illustrate the type of LDO predicted by WORMHOLE in difficult “edge cases”, we manually inspected a set of human-to-worm LDO predictions. Specifically, we examined genes that the WORMHOLE SVMs strongly selected (WORMHOLE RBHs with WORMHOLE Score > 0.75) but were missed by simple voting (Votes < 7, Vote Score < 0.25); 17 genes fit these criteria (Table 5). As a metric of sequence conservation, we conducted protein BLASTp for each query gene against the target genome, and each target gene against the query genome (Table 5). Of the 17 human genes queried, 5 had PANTHER LDOs in worm. In all five cases, WORMHOLE predicted the same worm gene as PANTHER. Four of these genes also were the BLASTp RBH between human and worm. In the remaining case (human gene *CPLX2*), both WORMHOLE and PANTHER identify the worm gene *cpx-1*, while a BLASTp of *cpx-1* against the human genome points to *CPLX1*.

**Table 5:** Human-to-worm WORMHOLE RBH predictions that received few votes (< 7, Vote Score < 0.25) but high WORMHOLE Scores (> 0.75).

WORMHOLE LDOs									Human -> Worm				Worm -> Human			
HumanGene	WormGene	PANTHERLDO	#Votes	Vote Score	WORMHOLEScore	BLASTpRBH?	Evolutionary Distance	Reciprocal Least Evolutionary Distance?	BLASTpBest Hit	BLASTpBit Score	Least Evolutionarily Distant Gene	Evolutionary Distance	BLASTpBest Hit	BLASTpBit Score	Least Evolutionarily Distant Gene	Evolutionary Distance
*PNISR*	*rsy-1*	*rsy-1*	6	0.203	0.862	yes	0.169	yes	*rsy-1*	88.2	rsy-1	0.169	*PNISR*	58.9	PNISR	0.169
*NPC2*	*heh-1*	*heh-1*	6	0.203	0.811	yes	0.200	no	*heh-1*	38.9	B0281.1	0.181	*NPC2*	38.9	NPC2	0.200
*NSMCE2*	*ZK1248*.*11*	*ZK1248*.*11*	6	0.203	0.811	yes	0.174	yes	*ZK1248*.*11*	43.9	ZK1248.11	0.174	*NSMCE2*	38.1	NSMCE2	0.174
*EIF3J*	*eif-3*.*J*	*eif-3*.*J*	5	0.154	0.791	yes	0.178	yes	*eif-3*.*J*	23.5	eif-3.J	0.178	*EIF3J*	23.5	EIF3J	0.178
*CPLX2*	*cpx-1*	*cpx-1*	6	0.203	0.824	no	0.118	yes	*cpx-1*	56.2	cpx-1	0.118	*CPLX1*	51.6	CPLX2	0.118
*SS18L2*	*ZK973*.*9*	none	6	0.203	0.849	yes	0.243	yes	*ZK973*.*9*	59.7	ZK973.9	0.243	*SS18L2*	59.3	SS18	0.146
*WASL*	*wsp-1*	none	6	0.203	0.888	yes	0.165	no	*wsp-1*	110	B0280.13	0.156	*WASF2*	43.9	WASL	0.165
*RAX2*	*ceh-8*	none	5	0.154	0.842	yes	0.145	no	*ceh-8*	113	ceh-53	0.141	*RAX2*	113	PRRX1	0.138
*BCL2L2*	*ced-9*	none	6	0.203	0.838	yes	0.193	no	*ced-9*	54.3	ced-9	0.193	*BCL2L2*	54.3	BCL2L1	0.178
*PDGFA*	*pvf-1*	none	6	0.203	0.824	yes	0.178	yes	*pvf-1*	50.8	pvf-1	0.178	*PDGFA*	49.3	PDGFA	0.178
*GPR139*	*B0334*.*6*	none	6	0.203	0.813	yes	0.176	no	*B0334*.*6*	77.8	B0563.6	0.173	*GPR139*	69.3	SSTR2	0.161
*SETD5*	*set-9*	none	6	0.203	0.802	yes	0.311	no	*set-9*	72	set-16	0.184	*SETD5*	73.6	ASH1L	0.195
*CCDC59*	*F10E9*.*11*	none	6	0.203	0.793	yes	0.215	yes	*F10E9*.*11*	39.7	F10E9.11	0.215	*CCDC59*	41.6	CCDC59	0.215
*SHB*	*Y87G2A*.*17*	none	6	0.203	0.788	yes	0.231	no	*Y87G2A*.*17*	120	shc-1	0.204	*SHB*	120	SHD	0.231
*HACD3*	*R10E4*.*9*	none	6	0.203	0.880	no	0.204	no	*hpo-8*	72.8	R10E4.9	0.204	*HACD3*	48.5	HACD4	0.157
*TNNI1*	*tni-4*	none	6	0.203	0.860	no	0.178	no	*unc-27*	63.5	unc-27	0.143	*TNNI2*	56.2	TNNI3	0.154
*RP11-343C2*.*11*	*vps-4*	none	5	0.154	0.779	no	0.045	no	*vps-4*	557	vps-4	0.045	*VPS4B*	627	VPS4B	0.039

In addition to the five gene pairs that the PANTHER LDOs called, WORMHOLE identified 12 novel LDOs that were not PANTHER LDOs (Table 5). Of these novel LDOs, 9 represent the BLASTp RBH for the query gene examined. In one of the three remaining cases, the human gene queried, *RP11-343C2*.*11*, overlaps nearly completely with another human gene, *VPS4A*. *VPS4A* is a paralog to the BLASTp RBH, *VPS4B*. This overlap suggests that *RP11-343C2*.*11* may be an artifact in the human genome used by the constituent algorithms predicting the gene pair. In another remaining case (human gene *TNNI1*), multiple duplication-post-speciation events have occurred between human and worm, and WORMHOLE identified one member of a closely related group of genes (*tni-4*) instead of another that is the BLASTp RBH (*unc-27/tni-2*).

We next examined evolutionary distance for each gene pair. In the case of human *CPLX1/2* and worm *cpx-1/2*, *CPLX2* is less evolutionarily distance from *cpx-1* than *CPLX1*, despite the failure of BLASTp to identify this pair as an RBH, suggesting that WORMHOLE is opting for the least divergent gene pair in this case. In contrast, the worm gene *heh-1* is identified as the WORMHOLE RBH, the PANTHER LDO, and the BLASTp RBH, but not the least evolutionarily distant gene (Table 5). Similarly, only 3 of the 12 novel WORMHOLE LDOs represent the reciprocal least evolutionarily distant gene between humans and worms. Which metric is “correct” in these cases is unclear, and phylogenetic reconstruction often does not provide additional insight. Many of these edge cases represent phylogenetic trees where gene duplication has occurred in both species more recently than the orthology-defining speciation event (e.g. *CPLX2/cpx-1* and *TNNI1/tni-4*). When this occurs, a single gene in one lineage will always be evolutionarily closer to all genes in the other lineage from the perspective of sequence divergence. For example, the *CPLX2* sequence has diverged less from both *cpx-1* and *cpx-2* than *CPLX1*. Other gene pairs belong to families with an even more complex and difficult to interpret evolutionary history with multiple speciation and duplication events (e.g. *HACD3/R10E4*.*9*). While the two genes in these complex families with the least sequence divergence are technically the LDO, the relationship between other family members, particularly when attempting to infer functional relationships from orthology, is ambiguous. In these cases, direct experimental examination is necessary to confirm functional relationships between orthologs. By considering consensus predictions from multiple prediction strategies, WORMHOLE provides a disciplined strategy for selecting genes prior to these analyses.

Taken together, these examples suggest that, with the PANTHER LDOs as reference and the additional information provided by the constituent algorithms, the WORMHOLE SVMs add clarity to difficult-to-distinguish edge cases where orthology is ambiguous based solely on an examination of available ortholog prediction strategies or voting-based meta-tools. They also help define the limits of the current SVM models around gene families with complex evolutionary history involving multiple speciation and duplication events that are not clearly resolved by current phylogenetic models.

### WORMHOLE web access

To provide convenient access to WORMHOLE LDO predictions, we developed a web tool that can be accessed publicly at http://wormhole.jax.org/. The web tool allows users to rapidly query the WORMHOLE database for LDO predictions between the six species, including options to manually define the WORMHOLE score threshold, exclude all but the highest scoring predicted LDOs for genes with multiple mappings, and select only WORMHOLE RBHs. Genome-wide ortholog predictions between each pair of species are also available for download.

## Discussion

The past two decades have seen the accumulation of a vast wealth of genetic information across thousands of species. On the heels of this accumulation, our ability to identify common genetic features between genomes has steadily improved, engendering dozens of methods for predicting orthologs based on sequence similarity, phylogenetic tree structure, and functional interactions. Here we introduce WORMHOLE, a novel application of machine learning to the problem of LDO prediction. In this tool we have taken advantage of the variety of available ortholog prediction strategies to develop a meta-tool that integrates predictions from many sources to specifically generate LDO predictions between six commonly used model organisms. The use of machine learning allows WORMHOLE to leverage the unique strengths of each method and the synergistic qualities between prediction methods to optimize performance and provide LDO predictions with higher confidence than other currently available methods, particularly when applied to predict LDOs between distantly related species.

### Multilayer machine learning approach

In developing WORMHOLE we have taken a supervised machine learning approach to LDO prediction that combines and augments current methods by adding a second layer that intelligently aggregates the predictions of many ortholog predictors into a compound LDO prediction. Multilayer methods are standard in machine learning and were originally biologically inspired. For example, the visual cortex of primates is organized into a hierarchy of neuron layers that successively capture higher order features of the visual field as the stimulus travels deeper into the brain. The earliest layers of the visual cortex capture relatively simple features of a scene like spots of relative brightness or darkness, intermediate layers aggregate these low-level features into object boundaries, while the highest layers relate these boundaries to semantic object categories stored elsewhere in the brain allowing for object recognition.

The multilayer structure of WOMRHOLE is analogous. In the case of WORMHOLE, the primitive features (e.g. bright and darks spots in the visual field) are represented by prior biological knowledge, such as sequence similarity, physical interactions between the protein products of genes, evolutionary distance between sequences, and known mutation rates as a function of taxonomy. The first layer of WORMHOLE—the 17 constituent algorithms—transforms these primitive features into intermediate features consisting of preliminary predictions of orthology between pairs of genes (analogous to the object boundaries in the visual cortex). These intermediate features individually are not always sufficient to distinguish LDOs, indeed the constituent algorithms do not intend to make such a prediction (see below), but each is a unique assessment of the many biological features that are relevant for such predictions. The second-layer aggregation operation integrates these preliminary predictions of the individual algorithms as input features for SVM classifiers, using the patterns in these features to recognize true LDOs (as the visual cortex recognizes objects from object boundaries) (Fig 1). This second layer is separated from the raw input data (genetic sequence) by the orthology predictions made by the constituent algorithms, combining them in an intelligent way to make LDO predictions.

We stress that the constituent algorithms do not intend to explicitly predict LDOs. Rather, they predict orthology by applying various statistical criteria to input data including phylogeny, sequence alignment, and/or functional annotation that are algorithm-specific. WORMHOLE uses the orthology calls of each algorithm as features that may be relevant to predicting LDOs. Indeed, LDOs are a specific and rather small subset of all orthologs. The extent to which any constituent algorithm’s ortholog or FEP predictions align with the PANTHER LDO reference set is a function of the methodology and the orthology definition used by that algorithm. Nevertheless, we can *treat* the orthology calls of the constituent algorithms as predictions of LDOs. If this assumption is not valid for a specific algorithm, the SVM will simply assign a low weight to that algorithm based on the observed poor performance of that algorithm at predicting PANTHER LDOs (e.g. Isobase, Fig 5A). From this point of view the constituent algorithms display wide variation in their precision and recall on the reference set; some are very conservative and precise, while others have high recall at the cost of calling many non-LDOs. On this basis we suspected that a simple voting strategy would be a useful heuristic for capturing likely LDOs by aggregating over a range of predictions and filtering out pairs that result from algorithm-specific errors or an overly broad orthology definition. Indeed, this voting strategy is enriched for LDO prediction compared to the constituent algorithms as it improves precision and recall over the constituent algorithms when predicting the PANTHER LDO set (Fig 2). More directly, the vote counts of PANTHER LDOs are significantly higher than non-LDOs (S6 Fig), demonstrating that voting is a discriminative criterion for LDO identification. While the performance improvement is species-dependent, voting achieves higher precision at a fixed value of recall (and vice versa) in nearly all cases.

The variation in precision and recall of the constituent algorithms demonstrates that giving each algorithm equal weight in the vote count is not optimal. Conservative algorithms that predict fewer orthologs but more often identify LDOs should be given higher weight. This raises the question of how to apportion weights to algorithms. One strategy would be to try to identify commonalities directly and construct weights “by hand”, but this runs the risk of incorporating our personal biases. Instead, we learned the weights from a training set of examples of true and false LDOs using the SVM algorithm (see Materials and Methods). The SVMs clearly outperform the simple voting by learning which algorithms are more trustworthy and giving them higher weight.

### “LDO-like” gene pair validation and functional cross-validation

In any machine learning application, the scope is defined exclusively by the training dataset. We trained our models on the PANTHER LDOs, a set of high quality LDO predictions. Because PANTHER LDOs represent a conservative set of closely related genes pairs, and because there exist edge cases for which evolutionary information becomes difficult to parse, we anticipated that the PANTHER LDOs were not comprehensive in identifying all true LDOs. Indeed, these edge cases increase in frequency for distantly related genomes that contain many duplication-post-speciation events in both lineages. PANTHER LDOs are very likely true positive LDOs, have high functional conservation, and they are more or less representative of true LDOs. However, because PANTHER LDOs are conservative, they are not comprehensive, making them a suitable reference set for predicting a larger set of LDOs. The central assumption of WORMHOLE is that we can learn a signature identifying true LDOs by inspecting the PANTHER LDOs. Our predictions are thus “PANTHER-LDO-like” as far as the input features to the SVM are concerned. We have employed four strategies to ensure that the WORMHOLE predictions are sensible: 1) nested cross-validation, which prevents overfitting on the training data, 2) estimation of evolutionary and sequence divergence between predicted LDOs, 3) prediction of known functionally equivalent proteins using a distinct set of high confidence FEPs (the FOSTA database), and 4) assessment of functional relatedness by measuring GO term conservation between predicted LDO gene pairs (using the community standard benchmarking service provided by QfO). Our results on the evolutionary and sequence divergence between WORMHOLE LDOs and RBHs are a direct test of “least divergence” between the predicted ortholog pairs. WORMHOLE LDOs and RBHs improve the PANTHER LDOs on these measures by: 1) expanding to a larger set of predicted LDOs without compromising the small divergence between predicted LDOs, and 2) excluding a subset of PANTHER LDOs that have significantly higher divergence than is typical of the PANTHER LDOs.

The tests of functional conservation and equivalence provide a completely independent assessment of the WORMHOLE predictions, but their results have to be interpreted with caution. First, as noted above, orthology is related, but not identical, to functional equivalence. Second, functional annotation of proteins is much less complete than predicted orthology. This is because sequence data are much more readily available than functional data and orthology can often be inferred with high confidence independent of any functional information. The SVMs perform better than voting and the constituent algorithms in predicting the FOSTA FEPs (S3 Fig). This relative comparison is what is important. The PR-curves for the SVMs tested on the FOSTA FEPs must be understood in light of the fact that many FEPs are likely to be missing from FOSTA. Likewise, when considering the conservation of functional annotations provided by QfO, there are many “missing” functional annotations, so performance has to be considered in a relative sense. The WORMHOLE RBHs have comparable functional similarity scores to the PANTHER LDO reference set, but WORMHOLE makes a substantial number of novel calls (Table 3, Fig 8 and S4 Fig). These novel calls are particularly important in distant species comparisons, where the methodology used to identify PANTHER LDOs is conservative. WORMHOLE employs complementary information not available to the PANTHER algorithm to improve confidence and expand the number of LDOs predicted. The functional cross-validation results suggest that WORMHOLE-predicted LDOs are sensible candidates.

Many of the WORMHOLE predictions are not one-to-one mappings, as required by the strict definition of an LDO. This can be interpreted simply as the expected “dead weight loss” of the machine learning strategy; the final model cannot reasonably be expected to perfectly predict the known LDOs and non-LDOs. An alternative interpretation is available when we observe that some LDOs will be less divergent from their non-LDO orthologs than others. Indeed, some genes will have multiple orthologs that are highly similar in both sequence and function, and selecting the LDO will amount to making an extremely fine distinction. These LDOs will be more difficult to separate using our strategy, but also much more functionally similar. The functional cross-validation shows that this is exactly what happens. Among the non-PANTHER LDOs (genes pairs in the WORMHOLE database, but not part of the PANTHER LDO dataset), a significant fraction lies within the larger PANTHER database (Fig 5A). These are the false positives that could not be reliably distinguished from true LDOs by the SVM during training. The functional cross-validation directly compares the WORMHOLE predictions to both the PANTHER LDOs and the full PANTHER set. The WORMHOLE predictions retain comparable scores to PANTHER LDO while calling many more pairs and producing better scores than other methods that call similar numbers of pairs. Simultaneously, WORMHOLE has higher performance than the full PANTHER set. We stress that this occurs purely as a side benefit of training an SVM to recognize LDOs from non-LDOs and not because WORMHOLE has explicitly included additional functional information beyond that contained in the first-layer algorithms.

Depending on the purposes of user, these functionally similar multiple mappings may be useful *per se*. A limitation inherent to the strict definition of an LDO as the *single* least diverged gene pair in an ortholog group is that it will necessarily fail to identify cases where a lineage-specific duplication results in redundant genes that are both functionally equivalent to the gene in the other species. Our functional data suggests that this is not a rare occurrence, as WORMHOLE predicts many multiple mappings that are enriched for functional conservation near the same level as the LDOs. However, there are two filters that a WORMHOLE user can use to sift through multiple hits to potentially identify the true LDO. First, within a family of hits the pair with the highest WORMHOLE score is likely to be the LDO (Fig 5C). An even stricter criteria is to select the gene pair with the reciprocal highest score (i.e. the WORMHOLE RBH), should it exist. However, some instances of multiple hits arise because the candidates have the exact same vote patterns, and hence the same WORMHOLE score. A second filter when considering multiple mappings is to use auxiliary criteria, e.g. highest-quality sequence alignment, independent of WORMHOLE to identify the LDO, which is beyond the scope of the WORMHOLE web tool.

### Cross-validation across species and general orthology

*A priori*, a highly tuned model to predict LDOs in one species pair might not have any predictive power for unrelated species. However, we find that a model trained on one species pair does perform well when applied to predict LDOs between other species pairs (Fig 3 and S3 Table). This strongly suggests that the WORMHOLE SVMs are identifying patterns in the constituent algorithm predictions that are indicative of LDO status in general and not just in the species pair used to train the model. This property points to broader applicability of the supervised machine learning framework and suggests that LDOs can be inferred in a species-independent manner. This is an intriguing prospect for future work.

### High quality candidate LDOs from WORMHOLE

An examination of novel LDO predictions made by WORMHOLE in gene pairs with ambiguous orthology status (Table 5) suggests that the WORMHOLE SVMs are able to parse non-intuitive information provided by the voting patterns in the constituent algorithms to provide clarity in distinguishing orthology. WORMHOLE identifies a number of novel LDOs in this realm, picking the BLASTp RBH in most cases. A few cases of disagreement between WORMHOLE and PANTHER or BLASTp indicate that there remains room for improvement by adding additional information or updating reference LDO sets in future iterations of WORMHOLE.

### Conclusions and future directions

WORMHOLE is the first machine learning meta-tool developed for the problem of predicting LDOs. We demonstrate the ability to improve LDO prediction using SVM classifiers. A key advantage to our approach is that it is a “meta-heuristic”, meaning that, in principle, any set of input algorithms can be used in Layer 1 and any user-preferred reference set and classification algorithm can be used in Layer 2. As more data become available and ever more sophisticated ortholog prediction tools are developed, the multilayer machine learning approach can grow to accommodate such innovations in the field. This work represents a starting point for several potential lines of future work. While WORMHOLE considers only the predictions of other orthology prediction methods, machine learning classifiers can accept any form of relational data for a given pair of potential orthologs that can be appropriately represented as input, allowing for consideration of information not implicitly captured in the constituent algorithms. In principle, future adaptations of WORMHOLE may include direct information about sequence similarity (e.g. alignment statistics), functional comparison (e.g. GO term conservation scores), or even more obscure biological information (e.g. relative expression levels in specific tissues). Beyond model systems, our results show that training a model on examples from one species pair generalizes well to other species pairs (Fig 3). It should be possible to use this property to make predictions in species not included in the design of WORMHOLE. Many current ortholog prediction projects make predictions for very large numbers of species. In principle, the machine learning framework can augment these predictions by, for example, training SVM models on a set of well-characterized and relevant models systems and using the predictions of the SVM models for less-characterized species. Some meta-tools (e.g. MOSAIC and MARIO) already use voting as a pre-processing step prior to sophisticated sequence-based analyses. Replacing simple voting with trained SVMs could supply candidates for sequence analysis at both a high level of sensitivity and specificity. The scope is only limited by availability of data and computational resources.

## Materials and Methods

### Source data

Ortholog and FEP datasets were acquired from the online repositories of each source database, in OrthoXML format when available. Web addresses, access dates, and version numbers for the 17 ortholog prediction datasets used to train WORMHOLE SVMs are provided in Table 1, and for all other source data in S6 Table. In building models, we were able to simply include all predictions generated by each tool under default settings in most cases. For EggNOG and Isobase, tool-specific considerations motivated additional effort.

#### EggNOG

EggNOG is a graph-based orthology prediction tool that builds orthologous groups (OGs) based on all-by-all similarity search followed by clustering using best-hit triangles between proteins from multiple species [17]. EggNOG groups proteins based on the MRCA within the context of the selected taxonomical group, allowing a hierarchy of OGs to be constructed based on the taxonomical range of the species included at each level. Two manually-curated categories are included that fall near the top of the hierarchy: Clusters of OGs (COGs), which include species across three kingdoms—Archaea, Bacteria, and Eukarya—and clusters of eukaryotic OGs (KOGs), which include a broad range of eukaryotic species. Non-supervised OGs (NOGs) form third category that represent automatically generated OGs that consider only groups of species within a specified taxonomical group (e.g. nemNOGs contain only nematode species). WORMHOLE considers EggNOG ortholog predictions based on COGs, KOGs, and Eukaryotic NOGs (euNOGs) as three independent categories (labelled EggNOG-COGs, EggNOG-KOGs, and EggNOG-NOGs in the remainder of this publication). The euNOGs were selected as the narrowest category that includes all six WORMHOLE species. In principle, the most appropriate taxonomic level to use should be dependent on the specific pair of species compared in a given query (e.g. for human-worm ortholog prediction, the bilaterial biNOGs should be used). As a consequence of only considering euNOGs, the input provided to WORMHOLE by the EggNOG-NOGs category will be less conservative for ortholog predictions between closely related species, such as humans and mice. The WORMHOLE classifiers will therefore give EggNOG-NOGs a lower weight in the final confidence score. Including a different taxonomic level for each pair of species would likely improve performance, but would add complexity to the construction of the database, and we opted to proceed with euNOGs across species for the initial presentation of WORMHOLE.

#### Isobase

Among the tools queried by WORMHOLE, Isobase is unique in its use of PPI networks to predict FEPs. Underlying the FEP prediction in Isobase is the IsoRankN algorithm, which uses protein sequence and interaction data to align PPI networks and generate clusters of functionally related proteins. The Isobase web tool allows genetic interaction (GI) data to be considered in addition to PPI data, employing the IsoRankN2 algorithm to simultaneously align PPI and GI networks, resulting in a set of FEP clusters distinct from that obtained considering PPI data alone. However, unlike the PPI data, species-wide ortholog predictions that include GI data are not made available for download on the Isobase website. Based on an informal survey of a number of proteins of interest using the Isobase web tool, we found that the FEP predictions that included GI data were clearly distinct from those including only PPI data. The pattern of ortholog predictions based on GI networks were not represented by any other selected prediction algorithm and thus potentially valuable as input to the WORMHOLE machine learning classifiers. This is particularly true in the cases of yeast and fruit fly, where a substantial quantity of genetic interaction data is available. In order to allow WORMHOLE to benefit from the unique aspects of this method, we applied IsoRankN and IsoRankN2 to generate updated ortholog predictions using current protein and genetic interaction data from 5 sources: the Biological General Repository for Interaction Datasets (BioGRID) [34], the Database of Interacting Proteins (DIP) [35], Human Protein Reference Database (HPRD) [36], the IntAct molecular interaction database [37], and the Molecular INTeraction database (MINT) [38]. Two independent sets of ortholog predictions were generated, one using IsoRankN and only considering PPI data (IsoRankN-PPI) and the other using IsoRankN2 to co-align PPI and GI data (IsoRankN2-GI). When compared, the three IsoRankN-based datasets (Isobase, IsoRankN-PPI, and IsoRankN2-GI) contained overlapping but distinct sets of ortholog predictions, so we opted to include all three in WORMHOLE for further analyses.

Including the three EggNOG categories, Isobase, and the two distinct IsoRankN categories, WORMHOLE considers input from 17 separate ortholog datasets. When the ortholog pairs predicted in these datasets are compared to the PANTHER LDOs, they display a wide range of precision and recall (Fig 2A).

#### Reference LDO dataset

PANTHER LDOs were selected as a reference set for high-confidence LDOs. PANTHER LDOs encompass all one-to-one ortholog predictions from the PANTHER database and include the single least diverged pair of genes from one-to-many and many-to-many ortholog predictions [7]. The PANTHER LDOs provides a strict, clearly defined set of high-confidence ortholog pairs that is suitable for training an SVM classifier.

#### Reference FEP dataset

A testing set of known FEPs was acquired from the Functional Orthologues from Swiss-Prot Text Analysis (FOSTA) database. The FOSTA database is provided in XML format and was converted to plain text using in-house Matlab scripts. The FOSTA database contains high confidence FEPs based on text analysis of Swiss-Prot annotations and thus represents an assessment of FEP at a high level of manual curation by experts. Each FOSTA FEP is anchored by a human protein; FEPs between non-human species were inferred by a co-annotation to a single human protein.

#### Reference identifiers

In order to compare data between sources, all protein and gene identifiers were mapped to a common set of reference identifiers. We selected Ensembl Gene IDs in Ensembl release 77 as the primary reference database. In cases where a gene or protein was not present in the Ensembl database, identifiers were mapped to National Center for Biotechnology Information (NCBI) Entrez Gene IDs or identifiers from organism-specific databases (e.g. WormBase for *C*. *elegans*). Mapped genes and ortholog predictions from all sources were used to build the WORMHOLE database.

### Voting, support vector machines, and cross-validation

As a first-order assessment of confidence in a given predicted ortholog pair we employ simple voting, a straightforward tally of the number of constituent algorithms that predict that pair. To improve upon simple voting, we applied machine learning to differentially weight the influence given to each algorithm based on its performance in predicting the reference LDOs. Specifically, let c denote a cLDO and let x^c^ ϵ {0, 1}^17^ denote the 17-dimensional binary vector of ortholog calls from each of the 17 constituent algorithms for c (i.e. $x_{i}^{c}$ denotes the 0/1 prediction of the i^th^ constituent algorithm). An SVM assigns a weight, w_i_, to predictions made by each constituent algorithm based on the individual performance of that algorithm at reproducing the PANTHER LDOs and defines a score for each cLDO, c:

$$raw\;SVM\;score(c)=\sum_{i=1}^{17}w_{i}x_{i}^{c}-b$$

where the sum is taken over the 17 constituent algorithms, w_i_ is the *weight* assigned to the i^th^ algorithm, and b is an *offset* that defines the boundary between positive and negative predictions. The parameters {*w_i_*,*b*} are learned from a set of labeled training examples. Note that if the offset is zero and all weights equal to one, then the SVM formula corresponds exactly to simple voting. Thus, the SVM is a *weighted* voting scheme where the weights are tuned to the training data. We fit the SVM classifiers using the R package “e1071”, which is available on the Comprehensive R Archive Network (http://cran.r-project.org).

In machine learning a key issue in model fitting is “overfitting”, i.e. setting the model parameters in such a way that the model performs well on the training data but fails to generalize to new data. The SVM has a single hyperparameter (i.e. a parameter that defines the fitting of the model, but not the model itself), called C that can be tuned to prevent overfitting. The parameter C defines the penalty for misclassifications and balances the fit to the data against generalizability [39]. For each combination of query and target species, we employed nested 10-fold cross-validation (nested CV) [40] to choose C. Nested CV splits the model selection process into an inner CV to select model parameters and an outer CV to estimate performance of the selection procedure. The outer CV, first randomly separates the data into 10 equal parts, trains the model on 9 parts, and tests the performance of the resulting model on the withheld part. The process is then iterated, withholding each 10% of the data once for testing. During each iteration of the outer CV, the model-training step is carried out using an inner CV. The 90% of the data used for training is further subdivided into 10 parts for standard CV. Within each inner CV iteration, C was chosen from a logarithmic vector, C ϵ {4^−2^, 4^−1^, 4^0^, 4^1^, 4^2^}, to maximize the average testing accuracy (fraction of correct classifications) on the 10^th^ inner CV parts. The inner-CV-tuned model is then tested on the 10% of the data that were held out for the outer CV. All assessments of generalization performance (precision, recall, harmonic mean) were estimated by their mean and standard error of mean over the 10 outer CV iterations. In this way, the inner CV ensures that the model parameters are not overfitting the idiosyncrasies of the training data, while the outer CV provides an estimate of the robustness of the model selection procedure when applied to novel data that were completely unseen by the model selection procedure during training.

Segregation of training data into training and test parts for cross validation requires care to ensure that each part is truly independent. Because there were many more negative examples than positive, we partitioned the two classes separately so that each part had the same proportion of positive and negative examples. We further ensured that all candidate ortholog pairs for a given query gene were assigned to a single part. We examined the effect of stratifying ortholog pairs into folds by gene family; however, while family-wise stratification resulted in a small increase in the variance of precision and recall across folds in the outer cross-validation, it did not affect either the overall quantitative performance of WORMHOLE or the qualitative conclusions reached in this work. Because the two strategies were qualitatively indistinguishable, we proceeded with the simpler, gene-wise stratification.

To compare SVM models between species-pairs (Fig 5B), we computed the Pearson correlation between the weight vectors, w, for each model. This correlation encodes whether or not the weight vectors assign high weight to the same set of constituent algorithms. For highly similar models this correlation is close to one, whereas for highly dissimilar models this correlation is close to zero.

### Performance assessment

As primary metrics of performance we evaluated recall (R) and precision (P). Recall is the fraction of the total number of correct ortholog pairs that are predicted by an algorithm, formally defined as:

$$R=\frac{T_{P}}{T_{P}+F_{N}}$$

while precision is the fraction of the total number of predictions made by an algorithm that are correct:

$$P=\frac{T_{P}}{T_{P}+F_{P}}$$

where *T*_*P*_ is the number of true positives, or the number of correct ortholog pairs predicted by an algorithm, *F*_*N*_ is the number of false negatives, or the number of correct ortholog pairs not predicted by an algorithm, and *F*_*P*_ is the number of false positives, or the number of incorrect ortholog pairs predicted by an algorithm. These values are calculated by comparing the pairs of orthologs predicted by each algorithm to the reference ortholog dataset for a given pair of query and target species. A single performance metric is often useful for comparing a large number of predictions. In these cases we used a related metric, the harmonic mean of precision and recall (*F*), defined as:

$$F=\frac{2PR}{P+R}$$

F provides a single measure that balances precision and recall.

### Scaling WORMHOLE confidence scores

The harmonic mean weighs P and R simultaneously and equally to summarize classification performance. A more flexible family of measures are the *β*-harmonic means defined by:

$$F_{\beta }=\frac{\left(1+\beta^{2}\right)PR}{\beta^{2}P+R}$$

The *β*-harmonic means are a family of measures that depend on a parameter, *β*, which balances the importance of recall relative to precision. The measure *F*_1_ is simply the harmonic mean (defined above). The measure *F*_0.5_ gives recall half the priority of precision, while *F*_2_ gives recall twice the priority of precision.

The raw confidence values returned by each SVM model (or voting) cannot be compared across species pairs because the assigned SVM weights are specific to each species pair. To allow such comparisons, we normalized the raw SVM scores to a scale that is directly linked to the performance of the model. Specifically, we identified the thresholds T within the raw scores for which the precision and recall at T maximizes F_β_ for β ϵ {0.125, 0.25, 0.5, 1, 2, 4, 8}. These thresholds are mapped onto the confidence scores 0.9375, 0.875, 0.75, 0.5, 0.25, 0.125, and 0.0625, respectively. We then interpolated that the map from raw SVM scores to confidence scores using monotonic Hermite cubic spline interpolation [41], which is implemented in the R function ‘splinefun’. Thus, the vote and SVM scores are scaled in such a way that applying a threshold of 0.5 (i.e. selecting all ortholog pairs with scores greater than or equal to 0.5) maximizes F for balanced precision and recall. Doubling *β* halves the distance toward 0.0/1.0 in the confidence scores. For example, a confidence threshold of 0.75 gives precision twice the weight of recall and a threshold of 0.875 gives precision four times the weight of recall. Conversely, a confidence threshold of 0.25 gives recall twice the weight of precision. We term the confidence score that scales simple voting the *Vote Score* and the confidence score that scales the raw SVM scores the *WORMHOLE Score*.

In a few cases, the score given to an ortholog pair differs depending on which species is used as query and which is used as target (e.g. a pair consisting of a human and worm gene may receive a different score if a human-to-worm query is made than when a worm-to-human query is made). This is a result of the way WORMHOLE is constructed, with a different SVM model used for each combination of query and target species. In order to harmonize scores with respect to direction of inquiry, the score given to each pair of orthologs by each Layer 2 WORMHOLE method was averaged between directions.

### Evolutionary distance and BLASTp bit scores

Evolutionary distance between genes was estimated for the longest protein encoded by each gene in a pair. Genome-wide protein sequences were obtained from Ensembl BioMart for each species [19]. All protein pairs between species were aligned using the *pairwiseAlignment()* function in the R package “Biostrings” [42], which implements quality-based alignment as described by Malde [43]. Evolutionary distance was calculated for each alignment using the *dist*.*ml()* function in the R package “phangorn” [44] using the BLOSUM62 substitution matrix. Both R packages are available on the Comprehensive R Archive Network (http://cran.r-project.org).

BLASTp bits scores and RBHs were determined by aligning each protein sequence against each target genome with NCBI BLAST+ (acquired from http://www.ncbi.nlm.nih.gov/blast; Table 1) using the following command options:

blastp-queryxxyy.fa-subjectxx.fa-outxx-yy-2.txt-outfmt6-max_hsps1-evalue1e-4

where “-query” and “-subject” specify input files in FASTA format, “-out” specifies the output file in text format, “-outfmt 6” requests BLASTp hits to be reported in a pairwise table with BLAST statistics, “-max_hsps 1” limits the output to a single report per matched protein pair, and “-evalue 1e-4” sets a maximum threshold on E-value for reported matches. The placeholders “xx” and “yy” indicated two letter abbreviations for species names (e.g. “ce” abbreviates *Caenorhabditis elegans*).

### Functional assessment using the Quest for Orthologs Benchmarking Service

The QfO Benchmarking Service tool accepts lists of predicted ortholog pairs and returns several measures of performance. We used this service to compare predictions made by WORMHOLE to those made by the PANTHER LDOs and the constituent algorithms for three performance criteria described by Altenhoff and Dessimoz [33], which are described briefly below. QfO provides several publicly available datasets for comparison, many of which are included as constituent algorithms in WORMHOLE. To minimize potential bias introduced by differences in the version of each dataset used in QfO vs. WORMHOLE, we independently ran each QfO performance metric on the set of ortholog pairs predicted in each constituent algorithm, as included in the WORMHOLE database. Each ortholog dataset (WORMHOLE, PANTHER LDOs, and constituent algorithms) was mapped to the QfO reference proteome and uploaded to QfO for analysis.

#### Gene-species tree discordance

Gene-species tree discordance is a measure of the disagreement between constructed phylogenetic gene and species trees. Because orthologs are gene pairs that diverge through speciation, the gene and species trees should have the same topology. Gene trees are constructed from the provided gene pairs using accepted species trees and the difference in topology between gene and species tree is quantified by computing the Robinson-Foulds (RF) split distance, defined as the normalized count of bipartitions in one tree but not the other. The tool also calculates the fraction of constructed gene trees that do not match the species tree.

#### Gene ontology term conservation

GO term conservation compares GO annotations between genes and assigns a similarity score. This test requires that both genes have at least one GO annotation. We report the mean Schlicker similarity, a similarity score that ranges from 0 (completely dissimilar) to 1 (identical). Schlicker similarity measures the concordance of GO term annotation between genes using a probabilistic formula related to the GO hierarchy [45].

#### Enzyme classification conservation

The enzyme classification (EC) conservation test functions similarly to the GO term conservation test, except that similarity is assessed between EC numbers rather than GO terms. As with GO term conservation, EC conservation requires that each gene in the predicted ortholog pair have at least one EC number, thus limiting the test to enzymes.

## Supporting Information

S1 Table Summary of ortholog datasets in the WORMHOLE database for each species combination. (XLSX)

S2 Table Performance of WORMHOLE SVMs, simple voting, and constituent algorithms at predicting PANTHER LDOs across the 10-folds of the outer cross-validation. WORMHOLE and voting values represent LDO predictions with scaled scores above the indicated threshold. SVM = support vector machine, P = precision, R = recall, F = harmonic mean of precision and recall.
(XLSX)

S3 Table Performance of WORMHOLE SVMs trained on one query and target species when tested on each other species pair. Values represent LDO predictions made by WORMHOLE SVMs with a WORMHOLE score threshold of 0.5. SVM = support vector machine, P = precision, R = recall, F = harmonic mean of precision and recall.
(XLSX)

S4 Table Jaccard index for pairs of constituent algorithms. As a measure of correlation between the predictions of the constituent algorithms, we computed the Jaccard index between each pair of constituent algorithm. The Jaccard index is defined as the number of orthologs that are predicted by both algorithms divided by the total number predicted by either algorithm. A Jaccard index of 1 indicates that the two algorithms completely agree, while a Jaccard index of 0 means that they never agree. The Jaccard indices vary substantially (mean ± st. dev. = 0.18 ± 0.11, min = 0.01, max = 0.53), indicating complex correlation structure between constituent algorithms.
(XLSX)

S5 Table Performance of WORMHOLE SVMs, simple voting, and constituent algorithms at predicting FOSTA FEPs. WORMHOLE and voting values represent LDO predictions with scaled scores above the indicated threshold. SVM = support vector machine, FEP = functionally equivalent protein, P = precision, R = recall, F = harmonic mean of precision and recall.
(XLSX)

S6 Table Source and access dates for PANTHER LDOs and other datasets used in this work. Source and access dates for the 17 ortholog datasets used to build WORMHOLE are provided in Table 1. LDO = Least Diverged Ortholog.
(XLSX)

S1 Fig WORMHOLE SVMs improve prediction of PANTHER LDOs over constituent algorithms and voting to a degree dependent on the evolutionary separation of the compared species. Precision-recall performance charts for PANTER LDO predictions across target species for genes queried in yeast **(A)**, worms **(B)**, fruit flies **(C)**, zebrafish **(D)**, humans **(E)**, and mice **(F)**. Points or lines represent the mean performance of the 17 constituent algorithms (black), voting (green), or WORMHOLE SVMs (blue) at predicting PANTHER LDOs across the 10 folds of the outer cross-validation (see Materials and Methods). Error bars and colored regions represent standard error of mean for precision and recall across folds. Lines are generated by sampling the complete range of possible threshold values for each confidence score type. Colored points indicate the performance for specified threshold values (blue numbers) on each line.
(PDF)

S2 Fig WORMHOLE identifies RBH pairs with a similar distribution of BLASTp alignment quality and evolutionary distance to the PANTHER LDOs and excludes low-scoring PANTHER LDOs. Box and whisker plots representing BLASTp Bit Score (A) or evolutionary distance (B) for alignments between longest protein isoforms for genes in each gene pair in the indicated ortholog dataset. Novel WORMHOLE RBHs are gene pairs predicted by the WORMHOLE RBH criteria that are not present in the PANTHER LDO training set. Excluded PANTHER LDOs are gene pairs in the PANTHER LDO training set that are excluded by the WORMHOLE RBH criteria.
(PDF)

S3 Fig WORMHOLE SVMs improve prediction of FOSTA FEPs over constituent algorithms and voting to a degree dependent on the evolutionary separation of the compared species. Precision-recall performance charts for FOSTA FEP predictions across target species for genes queried in yeast **(A)**, worms **(B)**, fruit flies **(C)**, zebrafish **(D)**, humans **(E)**, and mice **(F)**. Points or lines represent the mean performance of the 17 constituent algorithms (black), voting (green), or WORMHOLE SVMs (blue) at predicting FOSTA FEPs. Lines are generated by sampling the complete range of possible threshold values for each confidence score type. Colored points indicate the performance for specified threshold values (blue numbers) on each line.
(PDF)

S4 Fig WORMHOLE SVMs produce an expanded set of LDOs while maintaining functional similarity relative to PANTHER LDOs. Conservation of enzyme classification number (EC) between genes in enzyme gene pairs is plotted against the number of gene pairs contained with each dataset for constituent algorithms (black points), PANTHER LDOs (red lines), and WORMHOLE SVMs (blue lines). Points or lines indicate mean, and error bars or colored regions represent 95% confidence intervals, for Schlicker similarity in EC between genes (see Materials and Methods).
(PDF)

S5 Fig WORMHOLE SVMs produce LDOs representing an expanded set of gene trees while maintaining similar species-tree discordance relative to PANTHER LDOs. Mean discordance between gene and species phylogenetic trees represented by gene pairs in datasets for constituent algorithms (black points), PANTHER LDOs (red lines), and WORMHOLE SVMs (blue lines). Points or lines indicate mean, and error bars or colored regions represent 95% confidence intervals, for Robinson-Foulds (RF) distance between gene and species trees (see Materials and Methods).
(PDF)

S6 Fig PANTHER LDOs score dramatically higher on vote and SVM confidence scores. The number of votes **(A)**, Vote Scores **(B)**, or WORMHOLE Scores **(C)** received by PANTHER LDOs is dramatically higher than those received by gene pairs predicted by one or more of the constituent algorithms that are not in the PANTHER LDO set.
(PDF)
