# Supplementary material for: WORMHOLE: Novel Least Diverged Ortholog Prediction through Machine Learning
Source: PLoS Comput Biol. 2016 Nov 3;12(11):e1005182. doi: 10.1371/journal.pcbi.1005182 (PMC5094675; doi:10.1371/journal.pcbi.1005182)
Supplement: S1 Fig — Precision-recall performance charts for PANTER LDO predictions across target species for genes queried in yeast (A), worms (B), fruit flies (C), zebrafish (D), humans (E), and mice (F). Points or lines represent the mean performance of the 17 constituent algorithms (black), voting (green), or WORMHOLE SVMs (blue) at predicting PANTHER LDOs across the 10 folds of the outer cross-validation (see Materials and Methods). Error bars and colored regions represent standard error of mean for precision and recall across folds. Lines are generated by sampling the complete range of possible threshold values for each confidence score type. Colored points indicate the performance for specified threshold values (blue numbers) on each line. (PDF) [file pcbi.1005182.s007.pdf]

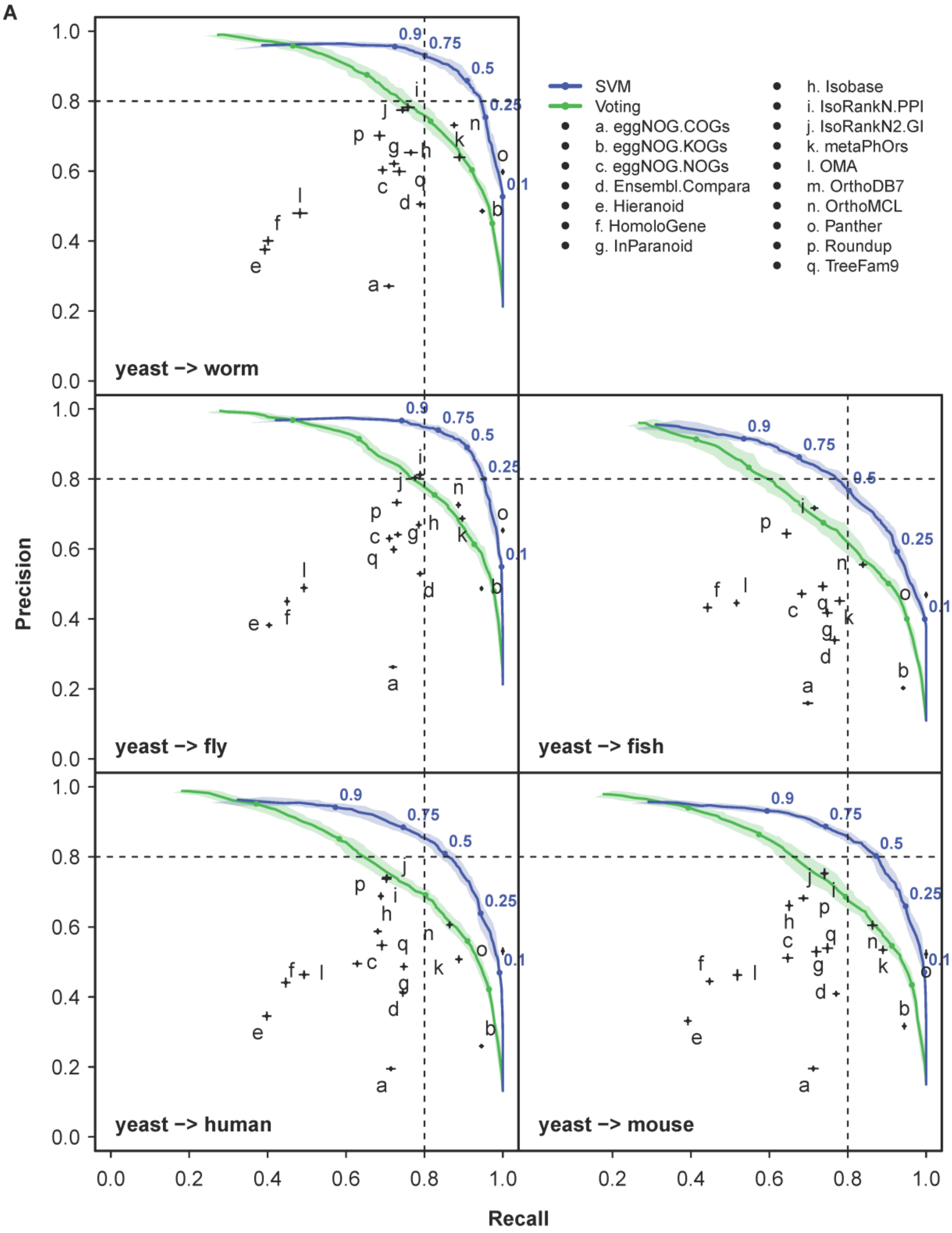

S1 Fig. WORMHOLE SVMs improve prediction of PANTHER LDOs over constituent algorithms and voting to a degree dependent on the evolutionary separation of the compared species. Precision-recall performance charts for PANTER LDO predictions across target species for genes queried in yeast **(A)**, worms **(B)**, fruit flies **(C)**, zebrafish **(D)**, humans **(E)**, and mice **(F)**. Points or lines represent the mean performance of the 17 constituent algorithms (black), voting (green), or WORMHOLE SVMs (blue) at predicting PANTHER LDOs across the 10 folds of the outer cross-validation (see Materials and Methods). Error bars and colored regions represent standard error of mean for precision and recall across folds. Lines are generated by sampling the complete range of possible threshold values for each confidence score type. Colored points indicate the performance for specified threshold values (blue numbers) on each line.

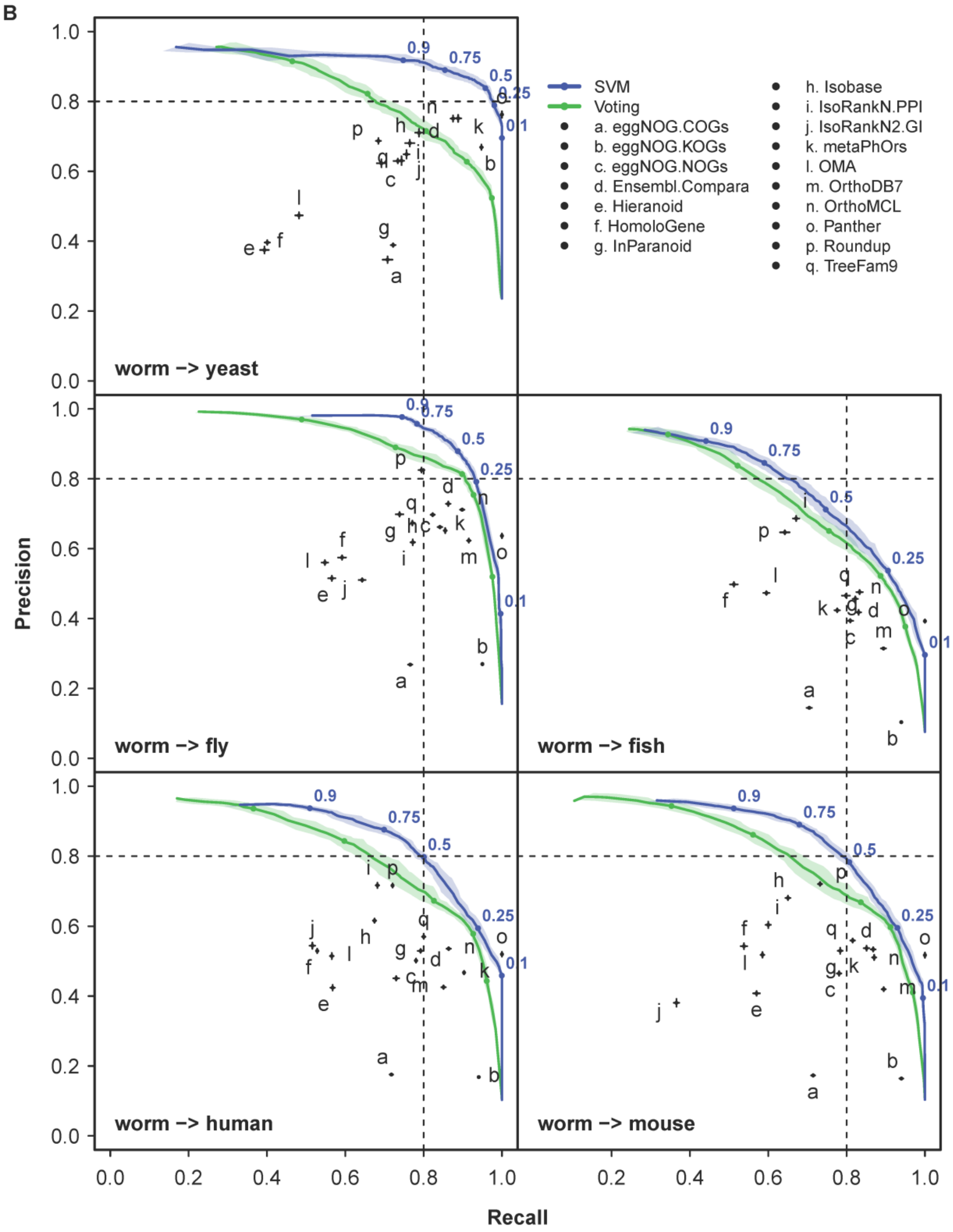

S1 Fig. Continued.

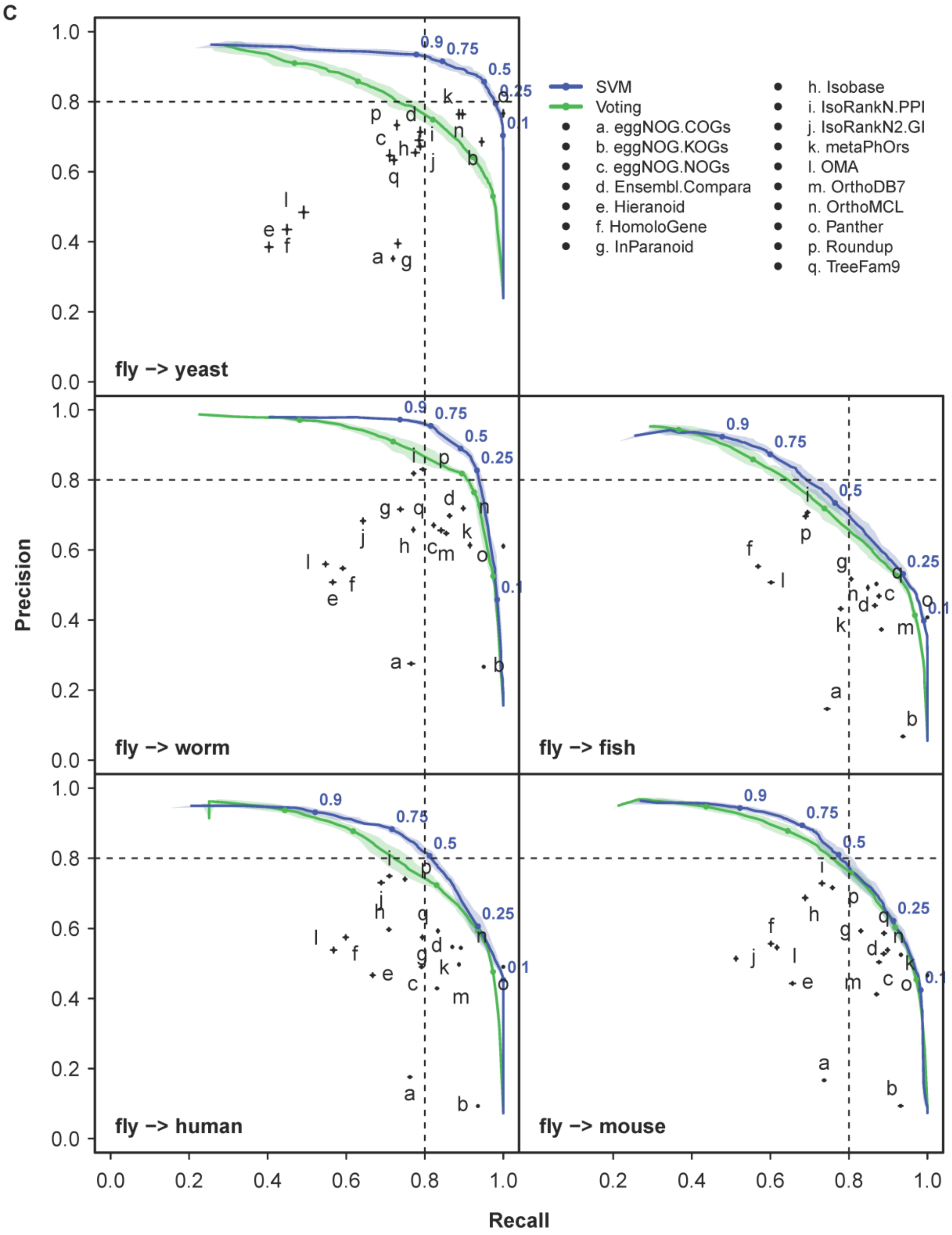

S1 Fig. Continued.

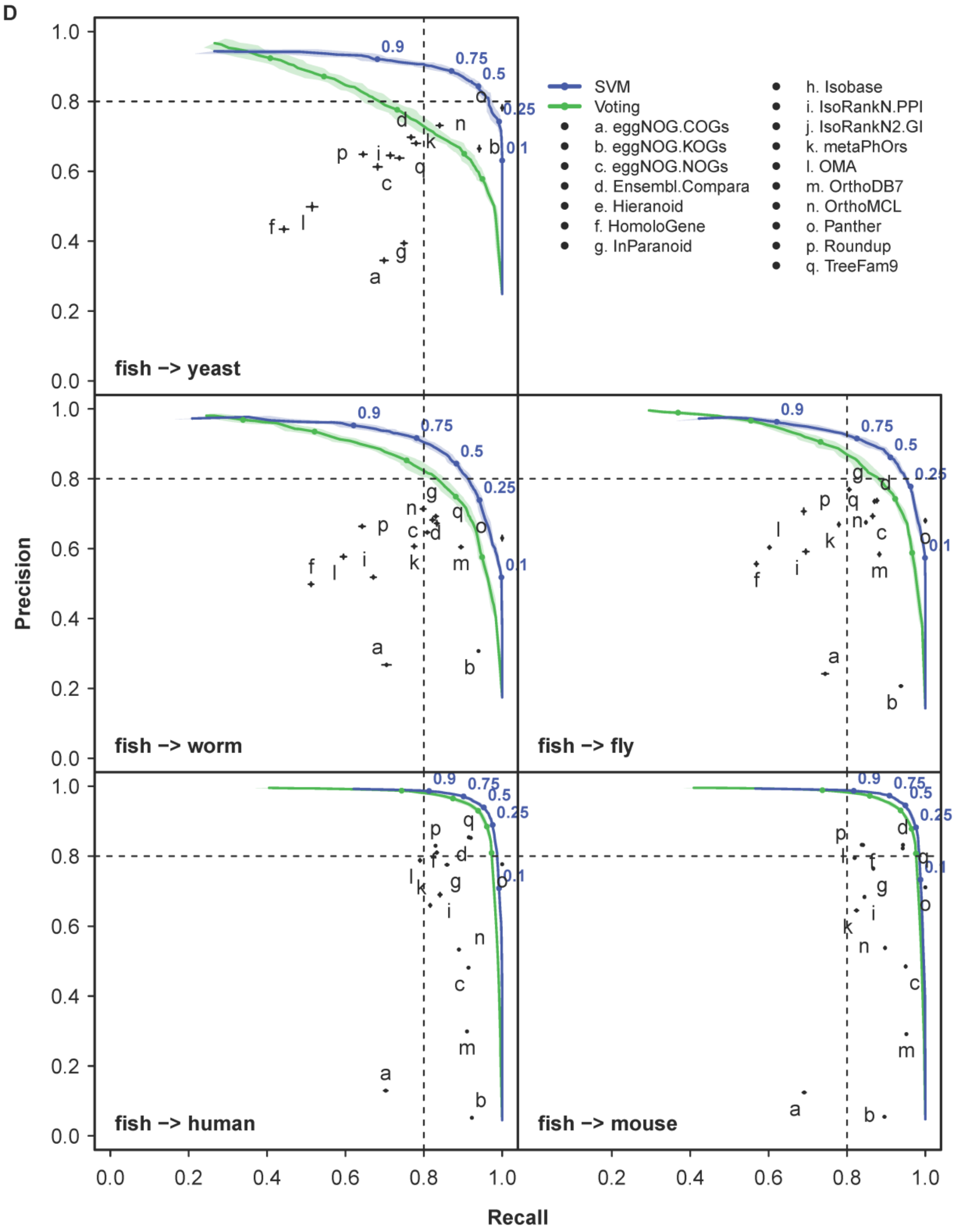

S1 Fig. Continued.

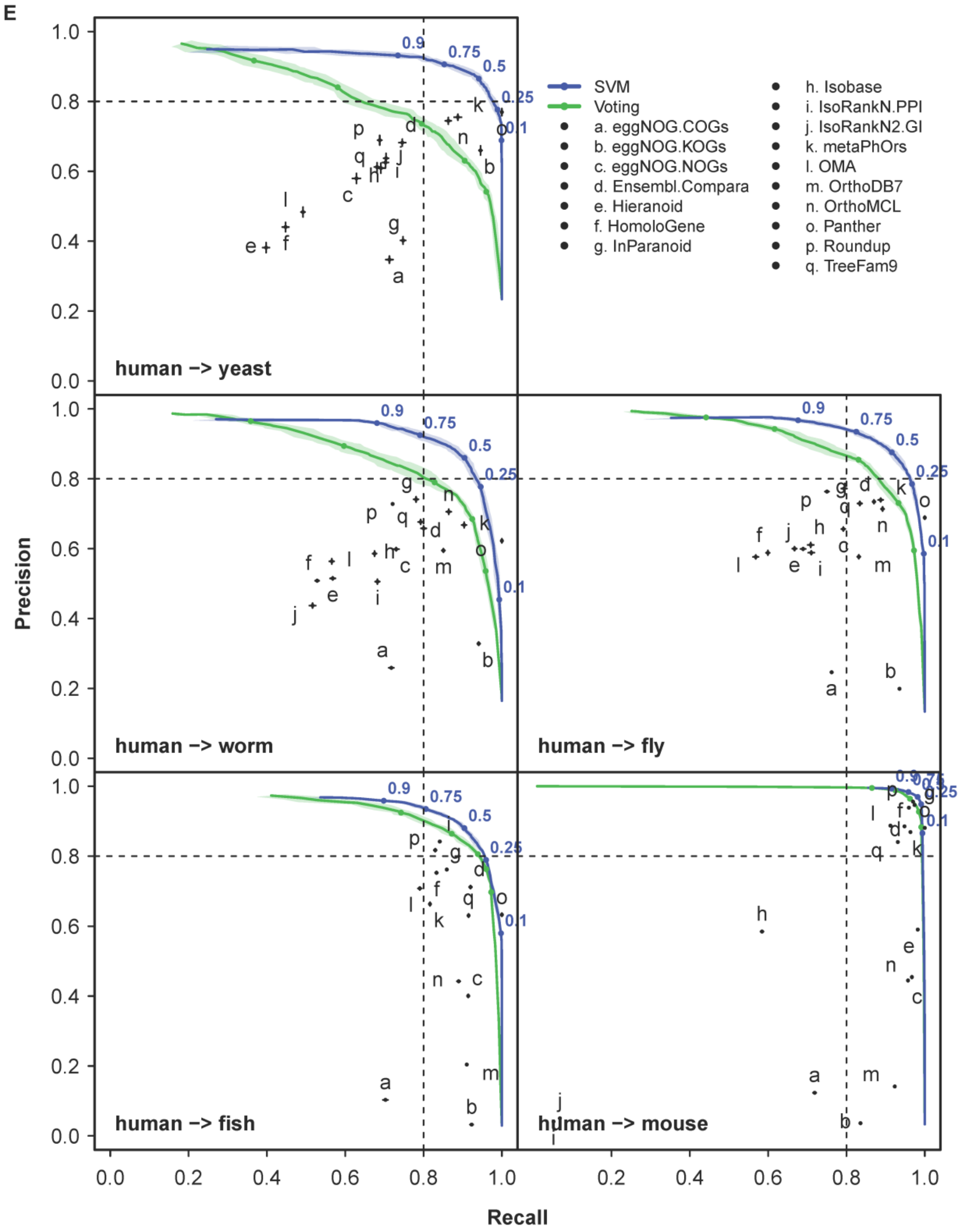

S1 Fig. Continued.

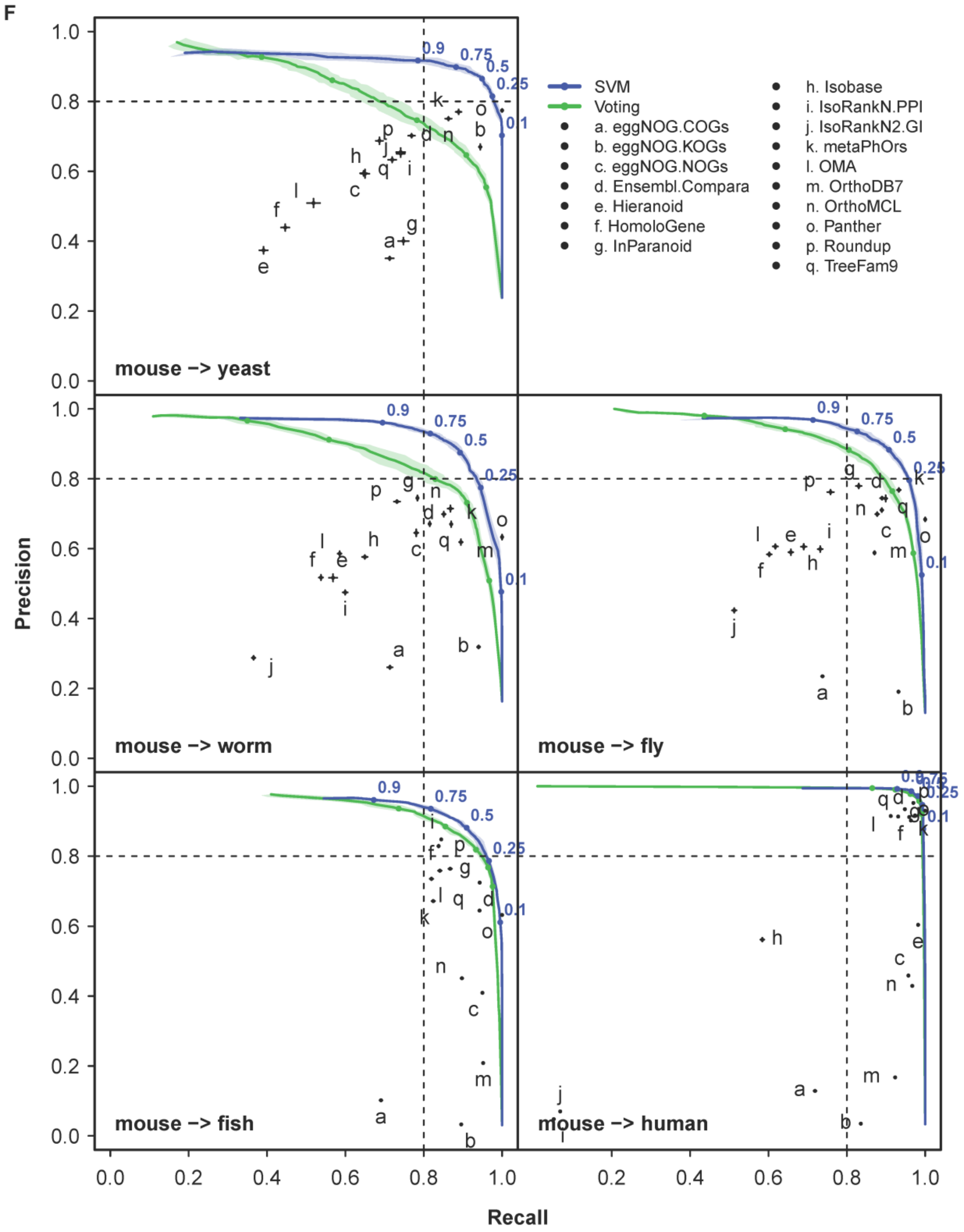

S1 Fig. Continued.
