# Supplementary material for: WORMHOLE: Novel Least Diverged Ortholog Prediction through Machine Learning
Source: PLoS Comput Biol. 2016 Nov 3;12(11):e1005182. doi: 10.1371/journal.pcbi.1005182 (PMC5094675; doi:10.1371/journal.pcbi.1005182)
Supplement: S2 Fig — Box and whisker plots representing BLASTp Bit Score (A) or evolutionary distance (B) for alignments between longest protein isoforms for genes in each gene pair in the indicated ortholog dataset. Novel WORMHOLE RBHs are gene pairs predicted by the WORMHOLE RBH criteria that are not present in the PANTHER LDO training set. Excluded PANTHER LDOs are gene pairs in the PANTHER LDO training set that are excluded by the WORMHOLE RBH criteria. (PDF) [file pcbi.1005182.s008.pdf]

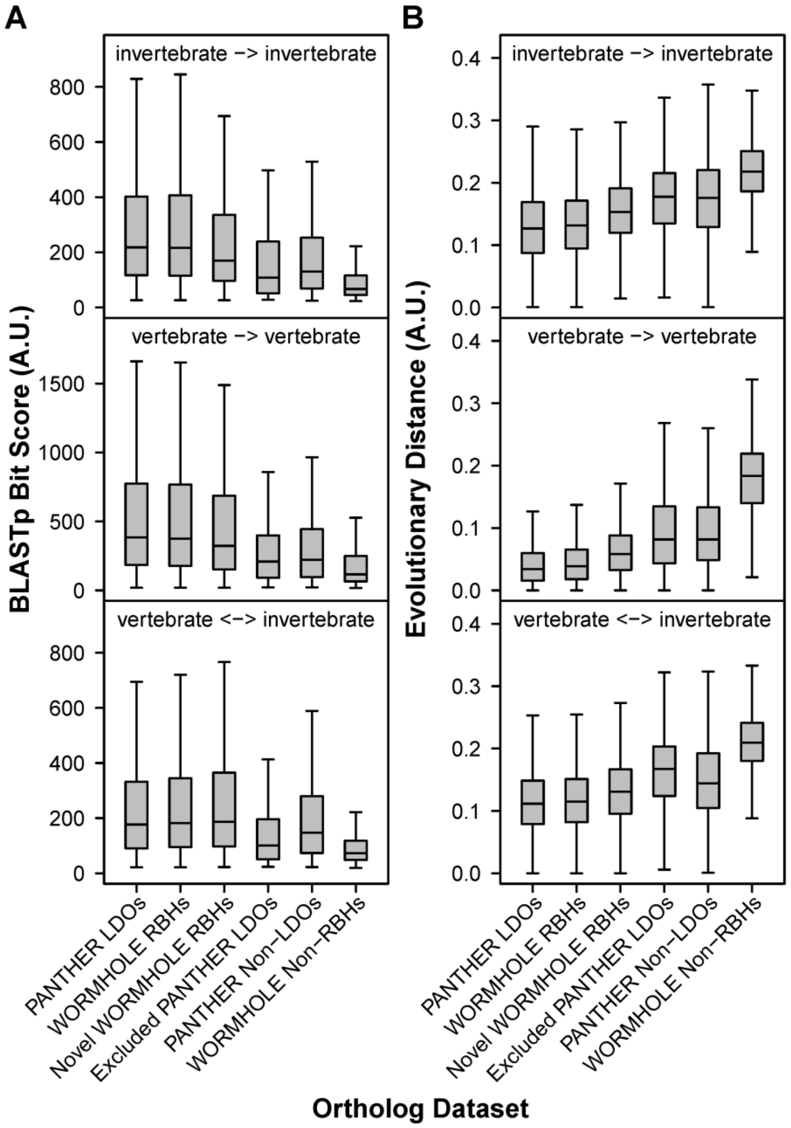

S2 Fig. WORMHOLE identifies RBH pairs with a similar distribution of BLASTp alignment quality and evolutionary distance to the PANTHER LDOs and excludes low-scoring PANTHER LDOs. Box and whisker plots representing BLASTp Bit Score **(A)** or evolutionary distance **(B)** for alignments between longest protein isoforms for genes in each gene pair in the indicated ortholog dataset. Novel WORMHOLE RBHs are gene pairs predicted by the WORMHOLE RBH criteria that are not present in the PANTHER LDO training set. Excluded PANTHER LDOs are gene pairs in the PANTHER LDO training set that are excluded by the WORMHOLE RBH criteria.
