# Supplementary material for: WORMHOLE: Novel Least Diverged Ortholog Prediction through Machine Learning
Source: PLoS Comput Biol. 2016 Nov 3;12(11):e1005182. doi: 10.1371/journal.pcbi.1005182 (PMC5094675; doi:10.1371/journal.pcbi.1005182)
Supplement: S3 Fig — Precision-recall performance charts for FOSTA FEP predictions across target species for genes queried in yeast (A), worms (B), fruit flies (C), zebrafish (D), humans (E), and mice (F). Points or lines represent the mean performance of the 17 constituent algorithms (black), voting (green), or WORMHOLE SVMs (blue) at predicting FOSTA FEPs. Lines are generated by sampling the complete range of possible threshold values for each confidence score type. Colored points indicate the performance for specified threshold values (blue numbers) on each line. (PDF) [file pcbi.1005182.s009.pdf]

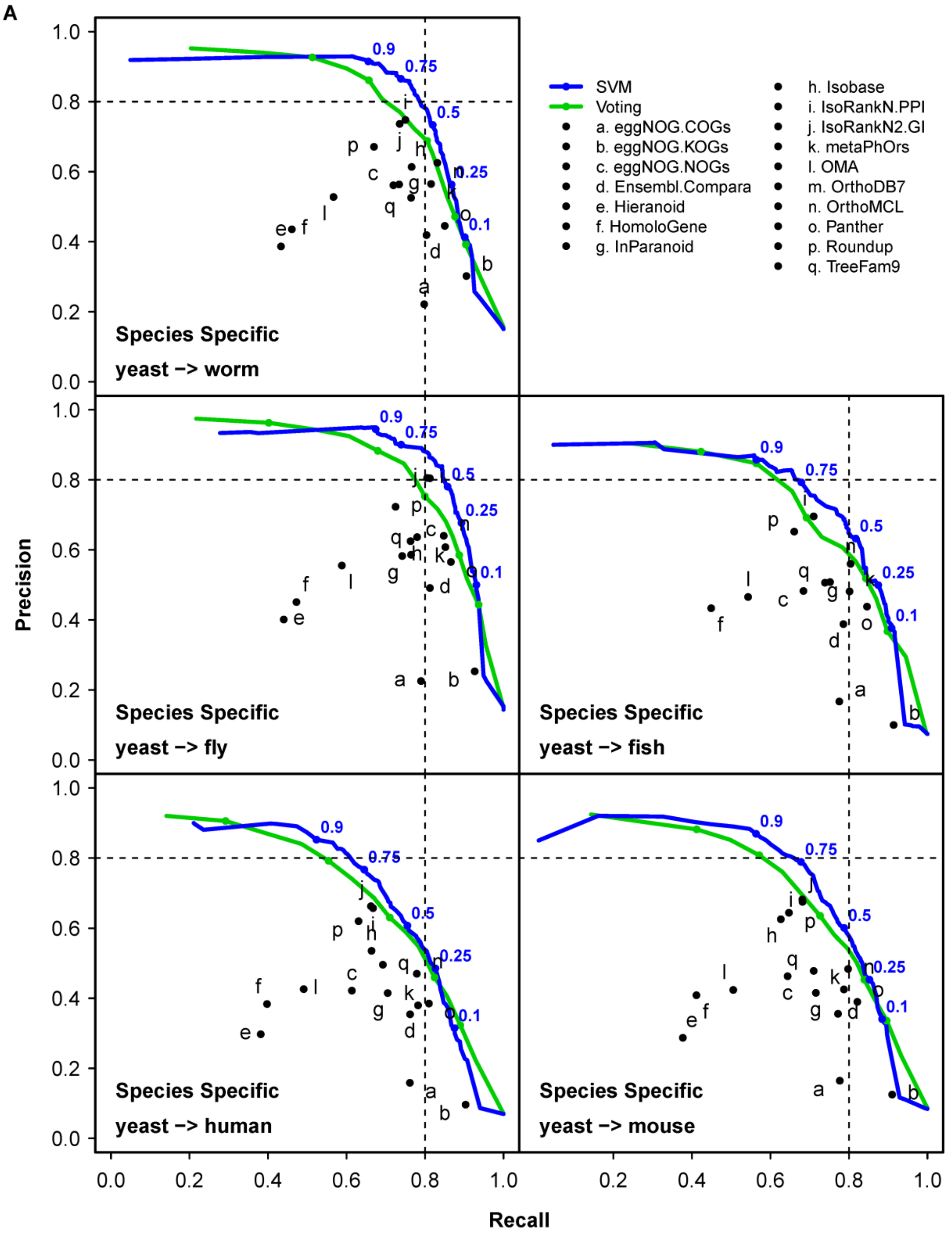

S3 Fig. WORMHOLE SVMs improve prediction of FOSTA FEPs over constituent algorithms and voting to a degree dependent on the evolutionary separation of the compared species. Precision-recall performance charts for FOSTA FEP predictions across target species for genes queried in yeast **(A)**, worms **(B)**, fruit flies **(C)**, zebrafish **(D)**, humans **(E)**, and mice **(F)**. Points or lines represent the mean performance of the 17 constituent algorithms (black), voting (green), or WORMHOLE SVMs (blue) at predicting FOSTA FEPs. Lines are generated by sampling the complete range of possible threshold values for each confidence score type. Colored points indicate the performance for specified threshold values (blue numbers) on each line.

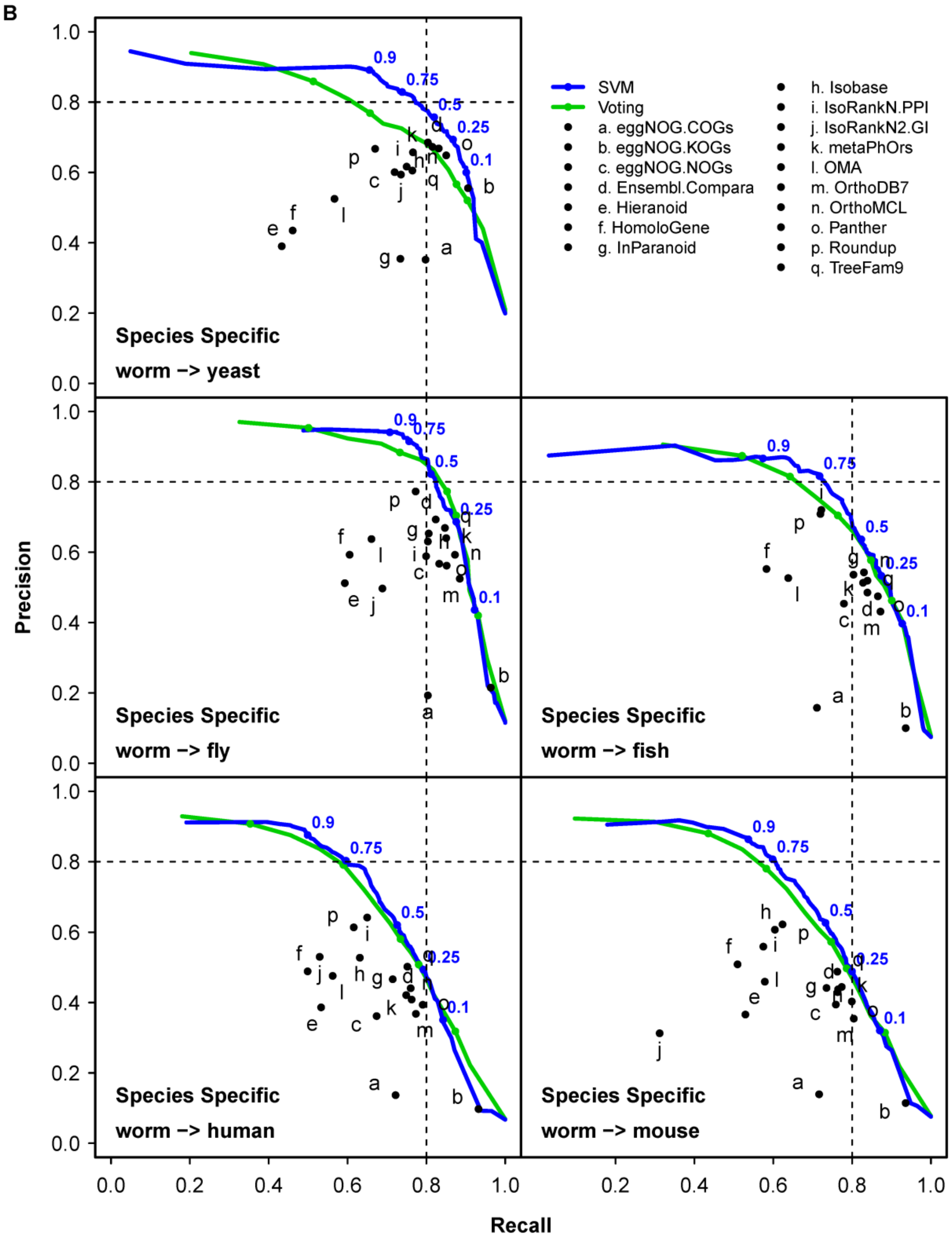

S3 Fig. Continued.

C

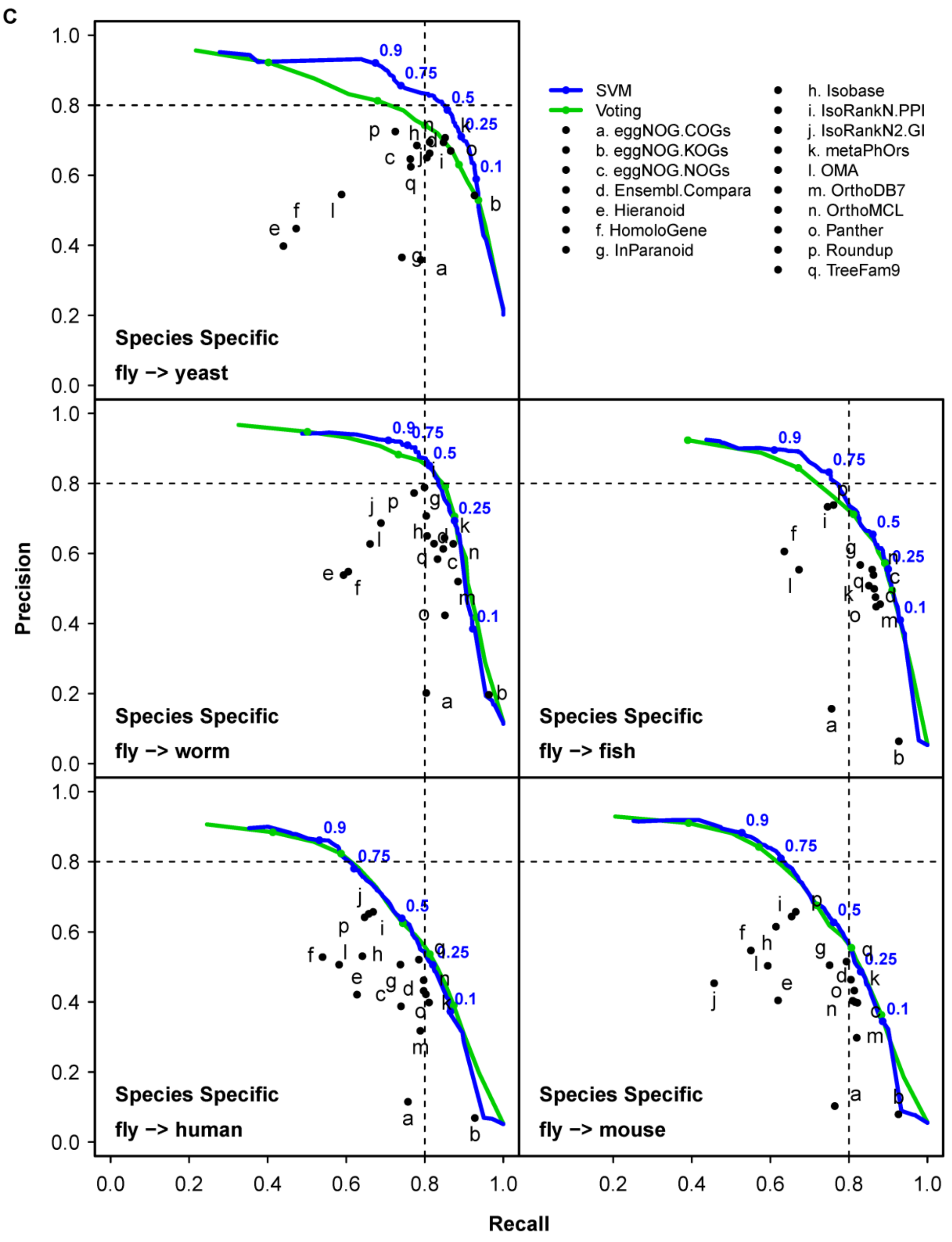

S3 Fig. Continued.

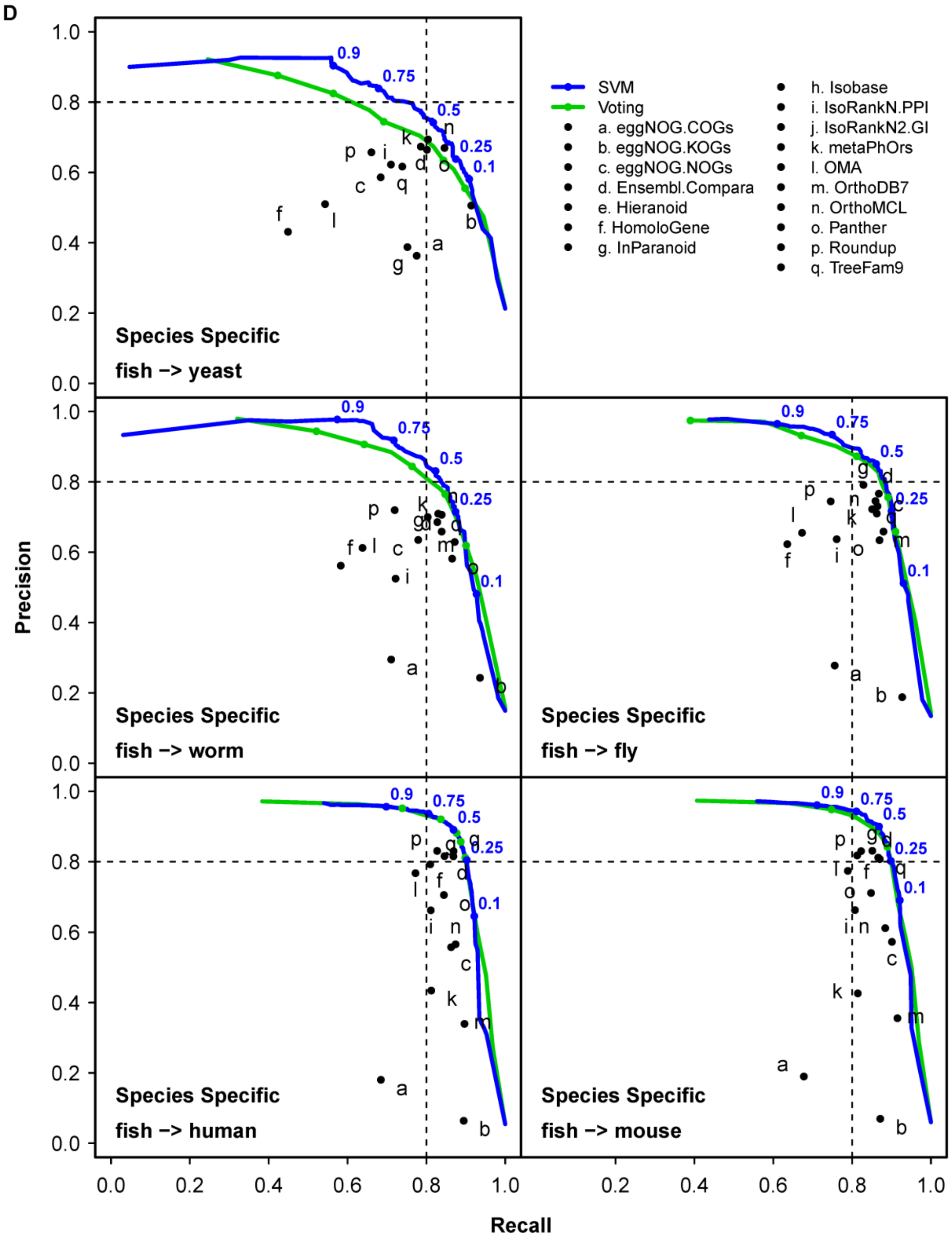

S3 Fig. Continued.

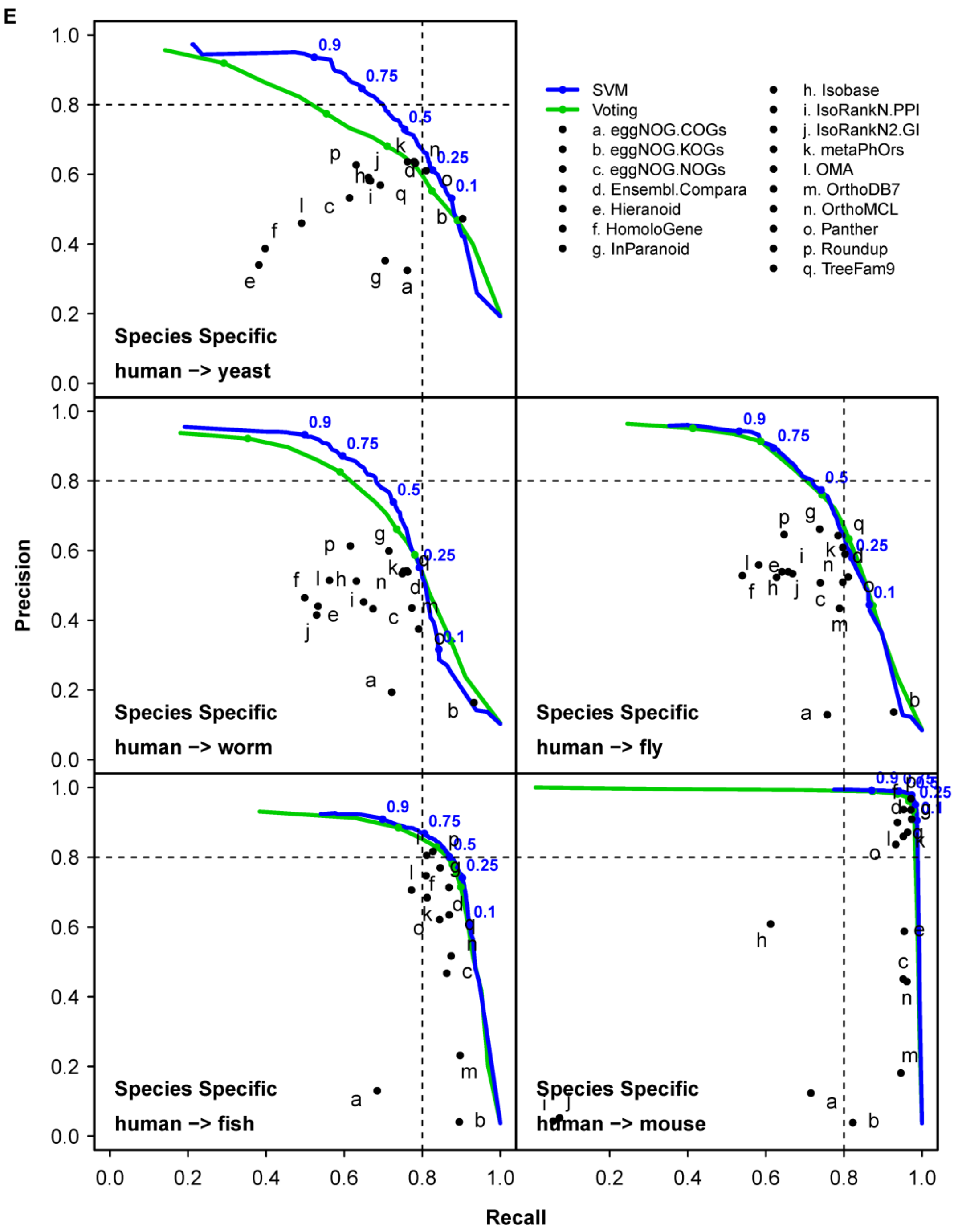

S3 Fig. Continued.

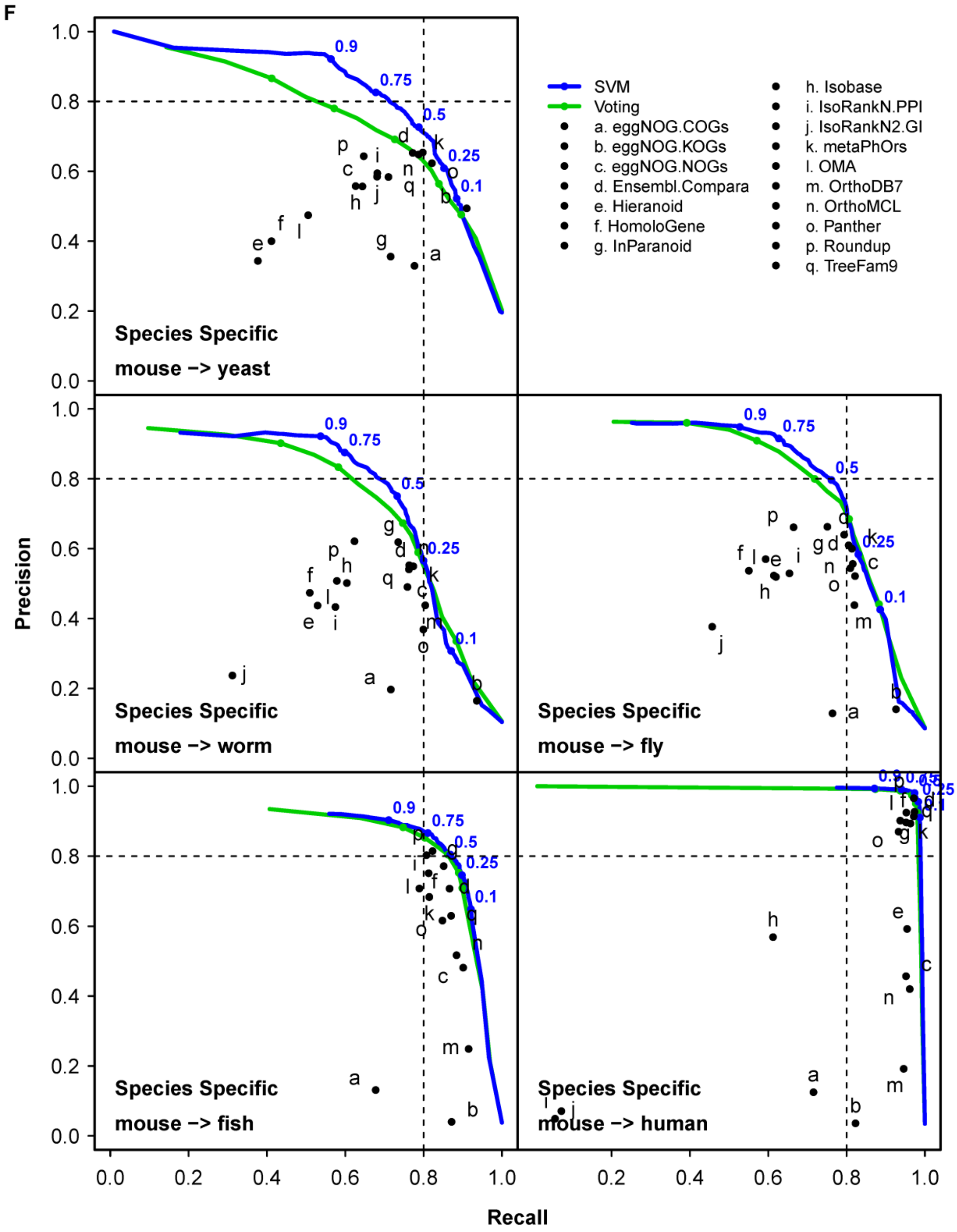

S3 Fig. Continued.
