# Supplementary material for: WORMHOLE: Novel Least Diverged Ortholog Prediction through Machine Learning
Source: PLoS Comput Biol. 2016 Nov 3;12(11):e1005182. doi: 10.1371/journal.pcbi.1005182 (PMC5094675; doi:10.1371/journal.pcbi.1005182)
Supplement: S4 Fig — Conservation of enzyme classification number (EC) between genes in enzyme gene pairs is plotted against the number of gene pairs contained with each dataset for constituent algorithms (black points), PANTHER LDOs (red lines), and WORMHOLE SVMs (blue lines). Points or lines indicate mean, and error bars or colored regions represent 95% confidence intervals, for Schlicker similarity in EC between genes (see Materials and Methods). (PDF) [file pcbi.1005182.s010.pdf]

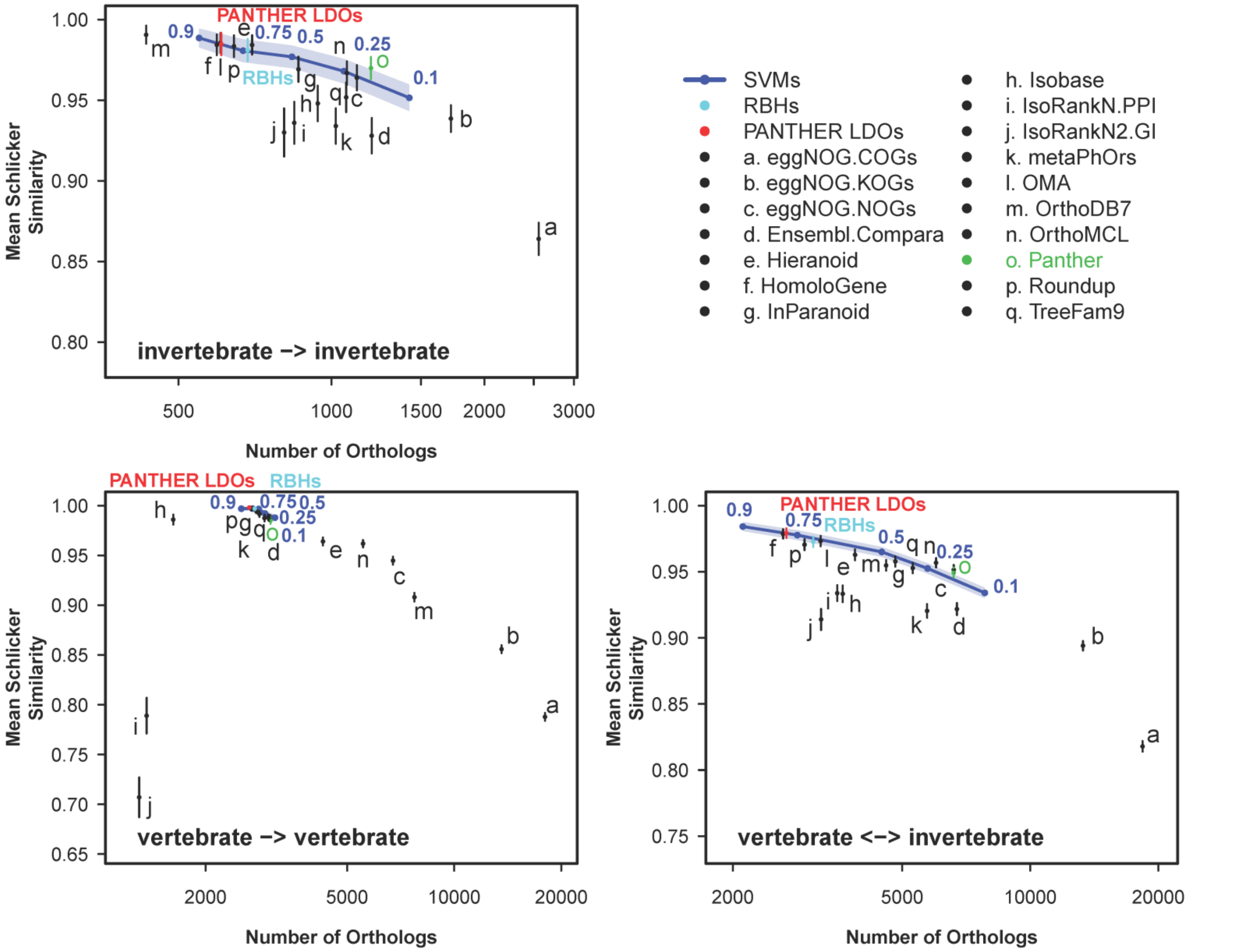

S4 Fig. WORMHOLE SVMs produce an expanded set of LDOs while maintaining functional similarity relative to PANTHER LDOs. Conservation of enzyme classification number (EC) between genes in enzyme gene pairs is plotted against the number of gene pairs contained with each dataset for constituent algorithms (black points), PANTHER LDOs (red lines), and WORMHOLE SVMs (blue lines). Points or lines indicate mean, and error bars or colored regions represent 95% confidence intervals, for Schlicker similarity in EC between genes (see Materials and Methods).
