# Supplementary material for: WORMHOLE: Novel Least Diverged Ortholog Prediction through Machine Learning
Source: PLoS Comput Biol. 2016 Nov 3;12(11):e1005182. doi: 10.1371/journal.pcbi.1005182 (PMC5094675; doi:10.1371/journal.pcbi.1005182)
Supplement: S5 Fig — Mean discordance between gene and species phylogenetic trees represented by gene pairs in datasets for constituent algorithms (black points), PANTHER LDOs (red lines), and WORMHOLE SVMs (blue lines). Points or lines indicate mean, and error bars or colored regions represent 95% confidence intervals, for Robinson-Foulds (RF) distance between gene and species trees (see Materials and Methods). (PDF) [file pcbi.1005182.s011.pdf]

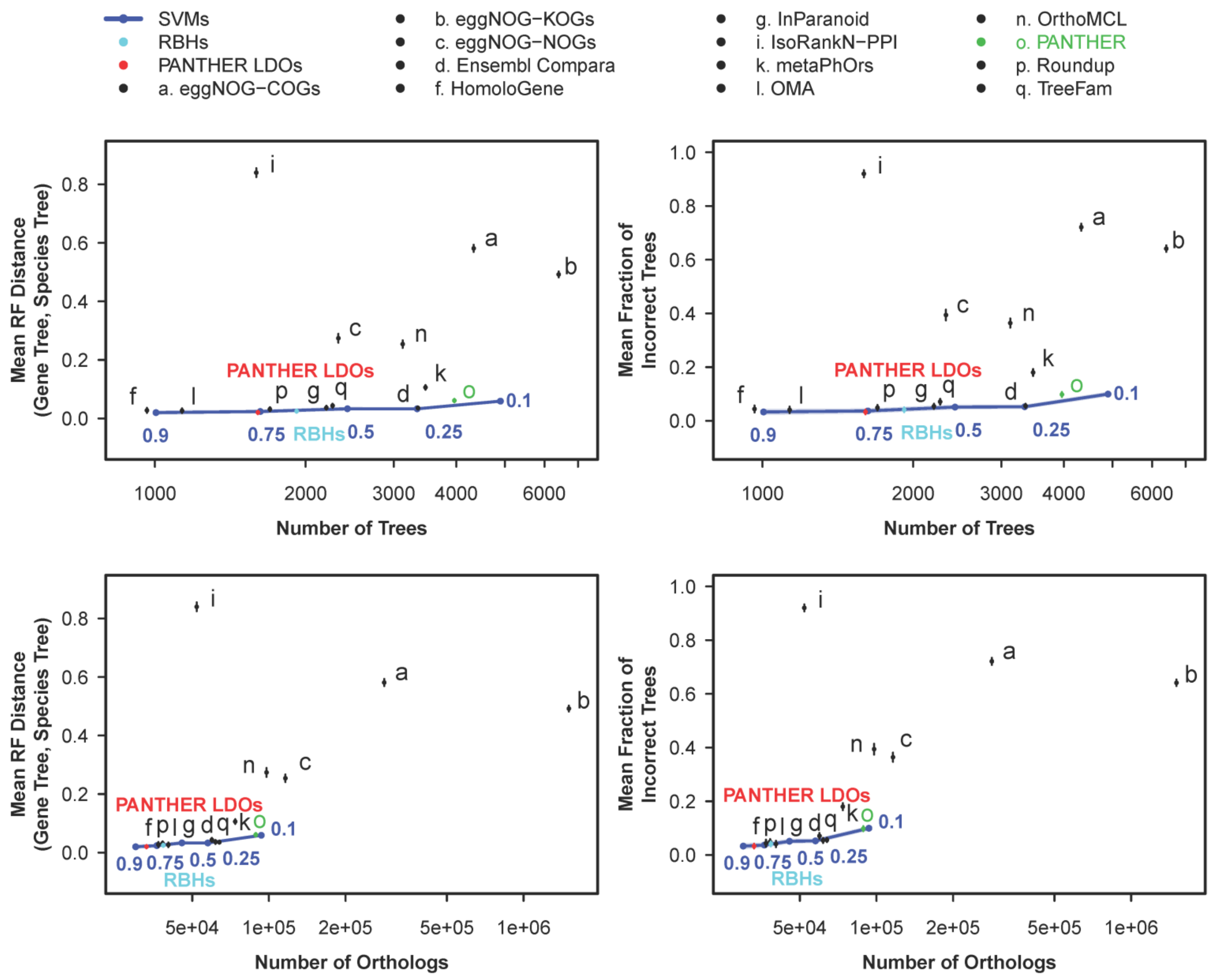

S5 Fig. WORMHOLE SVMs produce LDOs representing an expanded set of gene trees while maintaining similar species-tree discordance relative to PANTHER LDOs. Mean discordance between gene and species phylogenetic trees represented by gene pairs in datasets for constituent algorithms (black points), PANTHER LDOs (red lines), and WORMHOLE SVMs (blue lines). Points or lines indicate mean, and error bars or colored regions represent 95% confidence intervals, for Robinson-Foulds (RF) distance between gene and species trees (see Materials and Methods).
