# Supplementary material for: WORMHOLE: Novel Least Diverged Ortholog Prediction through Machine Learning
Source: PLoS Comput Biol. 2016 Nov 3;12(11):e1005182. doi: 10.1371/journal.pcbi.1005182 (PMC5094675; doi:10.1371/journal.pcbi.1005182)
Supplement: S6 Fig — The number of votes (A), Vote Scores (B), or WORMHOLE Scores (C) received by PANTHER LDOs is dramatically higher than those received by gene pairs predicted by one or more of the constituent algorithms that are not in the PANTHER LDO set. (PDF) [file pcbi.1005182.s012.pdf]

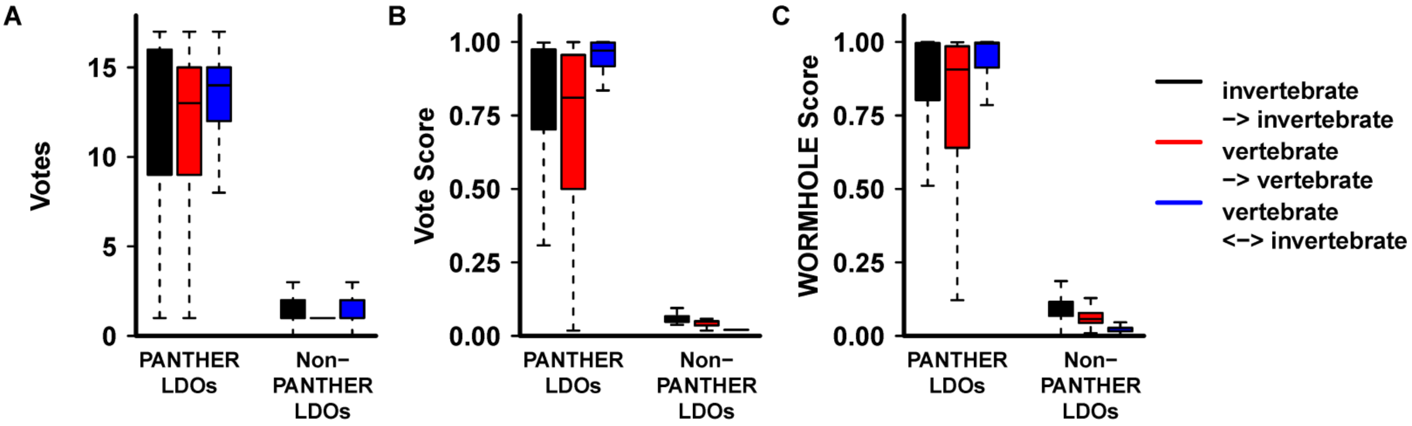

S6 Fig. PANTHER LDOs score dramatically higher on vote and SVM confidence scores. The number of votes **(A)**, Vote Scores **(B)**, or WORMHOLE Scores **(C)** received by PANTHER LDOs is dramatically higher than those received by gene pairs predicted by one or more of the constituent algorithms that are not in the PANTHER LDO set.
